# The duration of gastrin treatment affects global gene expression and molecular responses involved in ER stress and anti-apoptosis

**DOI:** 10.1186/1471-2164-14-429

**Published:** 2013-06-28

**Authors:** Linn-Karina M Selvik, Christina S Fjeldbo, Arnar Flatberg, Tonje S Steigedal, Kristine Misund, Endre Anderssen, Berit Doseth, Mette Langaas, Sushil Tripathi, Vidar Beisvag, Astrid Lægreid, Liv Thommesen, Torunn Bruland

**Affiliations:** 1 Department of Cancer Research and Molecular Medicine, Norwegian University of Science and Technology (NTNU), Trondheim N-7489, Norway; 2 Present adress: Weatherford Laboratories Inc., 16161 Table Mountain Parkway, Golden, Colorado 80403-1641, USA; 3 Department of Mathematical Sciences, Norwegian University of Science and Technology (NTNU), Trondheim N-7491, Norway; 4 Department of Technology, Sør-Trøndelag University College, Trondheim N-7489, Norway

**Keywords:** AR42J, Transcriptome, Temporal Profiles, Primary-and Secondary Genes, ERK1/2, AP-1, JUNB, UPR/ER Stress, Anti-apoptosis

## Abstract

**Background:**

How cells decipher the duration of an external signal into different transcriptional outcomes is poorly understood. The hormone gastrin can promote a variety of cellular responses including proliferation, differentiation, migration and anti-apoptosis. While gastrin in normal concentrations has important physiological functions in the gastrointestine, prolonged high levels of gastrin (hypergastrinemia) is related to pathophysiological processes.

**Results:**

We have used genome-wide microarray time series analysis and molecular studies to identify genes that are affected by the duration of gastrin treatment in adenocarcinoma cells. Among 403 genes differentially regulated in transiently (gastrin removed after 1 h) *versus* sustained (gastrin present for 14 h) treated cells, 259 genes upregulated by sustained gastrin treatment compared to untreated controls were expressed at lower levels in the transient mode. The difference was subtle for early genes like *Junb* and *c*-*Fos*, but substantial for delayed and late genes. Inhibition of protein synthesis by cycloheximide was used to distinguish between primary and secondary gastrin regulated genes. The majority of gastrin upregulated genes lower expressed in transiently treated cells were primary genes induced independently of *de novo* protein synthesis. This indicates that the duration effect of gastrin treatment is mainly mediated via post-translational signalling events, while a smaller fraction of the differentially expressed genes are regulated downstream of primary transcriptional events. Indeed, sustained gastrin treatment specifically induced prolonged ERK1/2 activation and elevated levels of the AP-1 subunit protein JUNB. Enrichment analyses of the differentially expressed genes suggested that endoplasmic reticulum (ER) stress and survival is affected by the duration of gastrin treatment. Sustained treatment exerted an anti-apoptotic effect on serum starvation-induced apoptosis via a PKC-dependent mechanism. In accordance with this, only sustained treatment induced anti-apoptotic genes like *Clu*, *Selm* and *Mcl1*, while the pro-apoptotic gene *Casp2* was more highly expressed in transiently treated cells. Knockdown studies showed that JUNB is involved in sustained gastrin induced expression of the UPR/ER stress related genes *Atf4*, *Herpud1* and *Chac1*.

**Conclusion:**

The duration of gastrin treatment affects both intracellular signalling mechanisms and gene expression, and ERK1/2 and AP-1 seem to play a role in converting different durations of gastrin treatment into distinct cellular responses.

## Background

Although the cellular response to one particular ligand is usually specific to a given cell type [1], some studies have shown that the duration [2-5] or concentration [4,6] of the external stimuli may also influence the transcriptional program and cell fate decision. Gene expression profiling in a pancreatic beta cell model treated with glucose and cAMP has shown that the majority of genes regulated in sustained treated cells were not regulated by transient (1 h) treatment, indicating that beta cells can produce drastically different transcriptional outputs in response to different durations of metabolic stimuli [2]. Glauser *et al*. [3] later showed that sustained elevated glucose promotes long-term phosphorylation of extracellular signal-regulated kinases (ERK1/2) followed by sustained changes in the activator protein 1 (AP-1 ) subunits composition as well as in AP-1 controlled gene expression. Hence, the authors suggested that in transiently (1 h) treated cells the ERK1/2 activation is too short to stabilize downstream gene expression. In human keratinocytes (HaCaT), sustained transforming growth factor beta (TGFβ) treatment induces a more persistent phosphorylation of the primary intracellular mediator SMAD2 than short pulses, and this may be critical for cell fate determination like cell growth arrest in this cell system [4]. Others have observed that a group of tumour necrosis factor (TNF) upregulated genes which remain at high levels in the sustained mode for 10 h, quickly returned to baseline if TNF was removed after 6 h [5]. However, in which way the duration of external signals affects molecular and biological responses has only been explored in a few cell systems and the mechanisms are still not well characterized.

The peptide hormone gastrin is the central regulator of gastric acid secretion and plays a prominent role in regulation of growth and differentiation of gastric and colonic mucosa [7,8]. Gastrin signals via the gastrin/cholecystokinin-2 (CCK2) receptor [9], and promotes a variety of cell or tissue specific outcomes including proliferation, survival, anti-apoptosis, differentiation and migration [7,10]. In normal physiological conditions, gastrin levels are transiently upregulated in response to a meal. Prolonged elevated blood levels of gastrin (hypergastrinemia) can occur as a consequence of e.g. atrophic gastritis or pharmacologic inhibition of gastric acid secretion, which interrupts negative feedback mechanisms on gastrin producing G-cells residing in the gastric mucosa [10]. Recently it was shown that the gastrin promotor can be activated by disease associated *Helicobacter pylori* strains via the EGFR/Raf/MEK/ERK cascade [11]. Both hypergastrinemia and mutational activation of the CCK2 receptor have been linked to development of neuroendocrine gastrointestinal tumours (carcinoids) [7]; and gastrin and CCK2R are reported to be upregulated in human pancreatic adenocarcinoma [12,13].

Since transiently increased gastrin levels have important physiological functions in the gastrointestine, while sustained high gastrin levels (hypergastrinemia) are related to pathophysiological processes [7,8,10,14], it is of interest to examine how the duration of gastrin treatment affects gene expression and molecular responses. We have therefore conducted the present study to examine how adenocarcinoma cells respond to transient *versus* sustained gastrin signalling and to identify characteristic differences between downstream biological responses. To do this, we analysed genome-wide time series data of gastrin-regulated gene expression which compared treatment in the transient (1 h) *versus* sustained (14 h) mode. We used data from additional gastrin response time series experiments to identify dependence on *de novo* protein synthesis related to transcriptional timing (early, delayed, late) for genes that are upregulated in the sustained mode (compared to untreated controls) and lower expressed in transiently treated cells. Enrichment analysis of genes differentially regulated in transient *versus* sustained gastrin signalling suggested that pathways related to ER stress, cell survival and anti-apoptosis were affected by duration of gastrin signalling. Indeed, several genes known to be involved in these pathways were expressed at lower levels in cells treated in the transient mode as compared to cells treated in the sustained mode. Furthermore, the anti-apoptotic effect of gastrin on serum starvation-induced apoptosis was dependent on sustained treatment, and our results indicate that the anti-apoptotic effect of gastrin involves PKC/ERK1/2 signalling. Sustained gastrin treatment induced prolonged ERK1/2 activation and elevated levels of the AP-1 subunit protein JUNB. The important role of ERK1/2 and AP-1 in converting different durations of gastrin treatment into distinct transcriptional responses was strengthened by our findings that knock down of JUNB reduced gastrin-mediated transcriptional activation of genes related to the ER stress and survival pathways. Overall, our work may contribute to a better understanding on how a cell deciphers the durations of gastrin induced signalling into specific cellular responses.

## Results and discussion

### Identification of genes that are differentially regulated by transient *versus* sustained gastrin signalling

**Figure 1:**
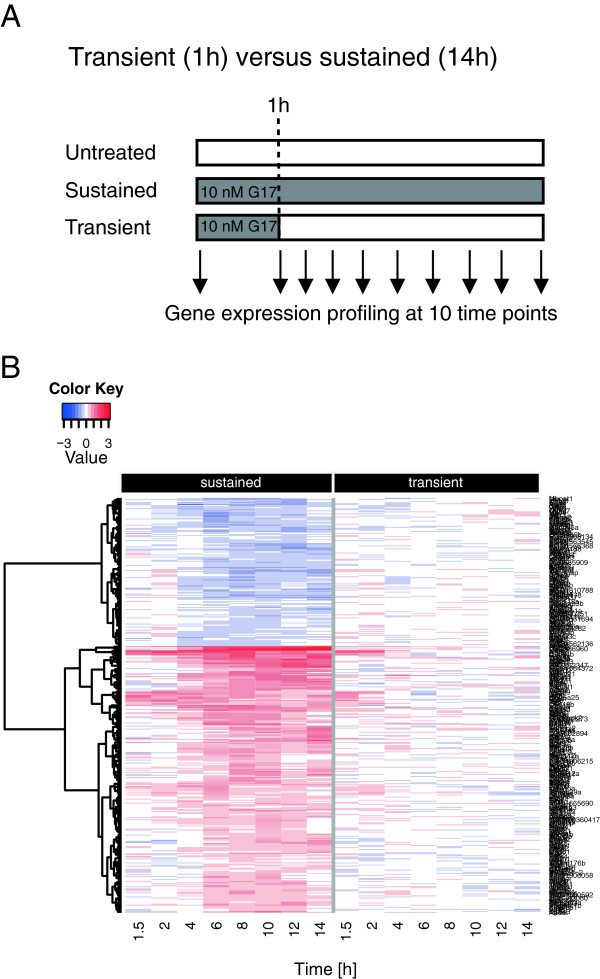
**Gene expression in transiently and sustained gastrin treated cells. A**: Schematic representation of stimulation protocol. Post confluent AR42J cells were serum starved for 20–24 h before 10 nM gastrin was added. Sustained treated cells (continuous presence of gastrin) were harvested at 9 different time points between 1 and 14 h. Transiently treated cells (gastrin removed after 1 h) were harvested at 8 different time points between 1.5 and 14 h. Untreated control cells were harvested at time point zero and throughout the time course (10 time points). **B**: Heat map of genes differentially expressed in transiently *versus* sustained treated cells. Temporal gene expression profiles of genes up- and down-regulated in sustained treated cells compared to untreated controls were hierarchical clustered and matched to temporal gene expression in transiently treated cells compared to untreated controls. The heat map shows average temporal gene expression in two independent experiments. The dendrogram is related to the hierarchical clustering of the first independent experiment with sustained treated cells.

To investigate the mRNA transcriptome in response to varying duration of gastrin treatment, we performed genome-wide microarray time series experiments in the adenocarcinoma cell line AR42J. We treated the cells in a sustained mode (14 h of continuous presence of gastrin) and in a transient mode (gastrin was removed after 1 h of treatment), as illustrated in Figure 1A. Statistical analysis of the time series gene expression responses identified 259 genes with lower and 144 genes with higher expression in cells treated with gastrin in transient *versus* sustained mode (Additional file 1: Table S1). The 403 differentially expressed genes were visualized in a cluster analysis heat map (Figure 1B). The upper part of the heat map shows that the 144 genes expressed at higher levels in the transient mode are downregulated by sustained gastrin treatment compared to untreated controls, while the 259 genes with lower expression in the transient mode (lower part of heat map) are upregulated by sustained gastrin treatment. The heat map further illustrates that differences in gene expression between cells treated in the transient and sustained mode were subtle at 1.5 h and 2 h, while substantial differences were apparent at time points after 2 h. The most striking overall trend for these 403 genes is that their transient mode expression levels after 4 h are similar to their levels in untreated cells, while they are clearly elevated or reduced in the sustained mode. These observations comply with our results from an earlier microarray 24 h time series study which showed that the regulation of several hundred genes was affected by gastrin signalling duration and that globally, gene expression levels returned earlier to baseline in cells subjected to transient (2 h) treatment compared to sustained treatment (Additional file 2: Accession number: E-MTAB-123). Thus, our results indicate that sustained gastrin signalling induces a transcriptional programme that differs significantly from the transcriptional response to transient gastrin signalling.

To search for molecular responses affected by the duration of gastrin treatment, the 403 genes exhibiting differential regulation in transiently *versus* sustained treated cells (Additional file 1) were subjected to pathway enrichment analysis using the MetaCore tool of GeneGo package [15]. We found that high scoring canonical pathway and networks were related to apoptosis-survival-development as well as to stress responses with a focus on the unfolded protein response (UPR), endoplasmic reticulum (ER)-stress, DNA damage and cell cycle (Table 1). Gene Ontology (GO) biological process enrichment analysis of the 259 genes with lower expression in transiently *versus* sustained treated cells also identified cellular responses to unfolded proteins as significantly enriched (Additional file 3: Table S2). Enriched GO processes associated with the 144 genes with higher expression in the transient mode were related to cell cycle phases and mitosis (Additional file 3: Table S3). Thus, it seems that sustained gastrin treatment is required for activation of genes involved in ER stress/survival (Additional file 3: Table S2), while many genes that are less downregulated by transient treatment encode proteins that promote cell proliferation (Additional file 3: Table S3).

**Table 1:** Enrichment analysis

	**P-values**	**Differentially expressed genes**
**Enrichment by GeneGo pathway maps**		
Apoptosis and survival_Endoplasmic reticulum stress response pathway	6,910E-05	ATF-4, GRP78, C/EBP zeta, IP3R1, HERP, DNAJC3, ERP5
Reproduction_GnRH signalling	7,392E-05	PER1, JunB, ATF-3, EGR1, IP3 receptor, Dynamin-1, HDAC5, c-Fos
DNA damage_ATM / ATR regulation of G2 / M checkpoint	1,262E-04	Wee1, Cyclin B, GADD45 alpha, Cyclin A, CDK1 (p34)
Development_Growth hormone signalling via PI3K/AKT and MAPK cascades	1,485E-04	JunB, C/EBP zeta, 4E-BP1, EGR1, C/EBPbeta, c-Fos
Development_Hedgehog and PTH signalling pathways in bone and cartilage development	6,180E-04	EGR1, Smoothened, Ihh, Cyclin A, c-Fos
**Enrichment by GeneGo process networks**		
DNA damage_Checkpoint	1,639E-05	CIA/ASF1, PCNA, Wee1, Cyclin B, GADD45 alpha, Cyclin B2, ATF-3, Cyclin A1, 14-3-3 theta, Cyclin A, CDK1 (p34), CDK6, 14-3-3
Cell cycle_Mitosis	5,376E-05	Tubulin beta, Kid, PARD6A, Wee1, Cyclin B, CSE1L, Cyclin B2, TTK, CAS-L, MKLP1, Dynamin-1, Cyclin A, CDK1 (p34), PARD6, Dynamin
Protein folding_Response to unfolded proteins	3,657E-04	ATF-4, ERp44, GRP78, HSP70, Calreticulin, HERP, DNAJC3, SELS

Canonical pathways and networks most significantly associated with the 403 genes differentially expressed (p<0.05) in transient *versus* sustained gastrin treatment shown in the heat map in Figure 1B and in Additional file 1. The 5 pathways and 3 networks with lowest p-values are shown as determined by the MetaCore tool of GeneGo package [15]. Enrichment analysis by GO process is found in Additional file 3: Tables S2 and S3.

**Figure 2:**
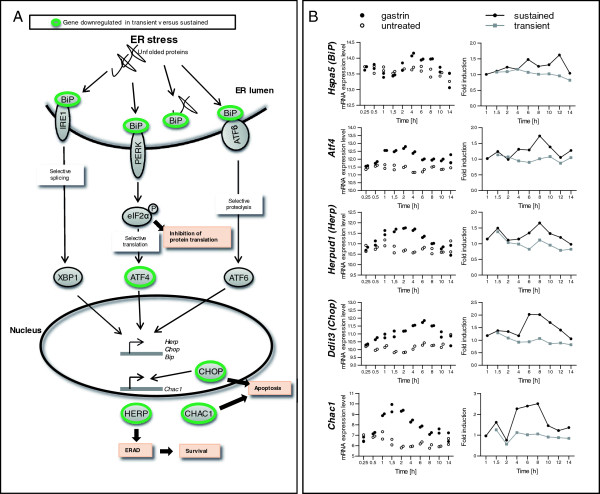
**Genes involved in the unfolded protein response (UPR) differ in transiently versus sustained gastrin treated cells. A**: Schematic presentation of the three signalling pathways initiated by the stress sensors IRE1, PERK and ATF6 in the UPR. The green circles indicate genes differentially expressed in transiently *versus* sustained gastrin treated cells. IRE1, PERK and ATF6 are associated with the protein chaperone BiP (HSPA5/GRP78) in their inactive state. In response to stress, unfolded proteins accumulate and bind to BiP, leading to release and activation of the three stress sensors and activation of their respective pathways: IRE1 activates and initiates nonconventional splicing of Xbp1 mRNA. PERK phosphorylates eIF2α leading to a general attenuation of translational initiation and a selective induction of ATF4 translation. ATF6 transits to the Golgi where it is cleaved to yield a cytoplasmic fragment which moves into the nucleus. XBP1, ATF4 and ATF6 activate a wide variety of UPR target genes, including *BiP*, *Chop*, *Herp* and *Chac*1 [16,20,21]. **B**: Data from two independent time series microarray experiments showing time profiles for UPR genes differentially expressed in transiently *versus* sustained gastrin treated cells. Left panels: The data were extracted from a time series experiment where sustained gastrin treated cells were harvested at 10 different time points between 15 min and 14 h. The samples from untreated control cells were harvested at time zero and throughout the time course (11 time points). The mRNA expression level for untreated (open dots) and sustained gastrin treated (black dots) cells are shown as normalized log2-transformed signal intensities (N=2). Right panels: Gastrin induced gene expression in transiently (grey lines) and sustained (black lines) treated cells (stimulation protocol presented in Figure 1). The data is shown as mean fold induction relative to untreated cells at the same time point (N=2).

ER stress/UPR is increasingly recognized to play a role in tumourigenesis as well as in cell homeostasis [16-18]. Thus, our novel findings that gastrin *i*) regulate ER stress/UPR genes and *ii*) does so in a signal duration-specific manner are highly interesting. Depending upon the duration and the degree of ER stress, the UPR can provide either survival signals by activating adaptive and anti-apoptotic pathways, or death signals by inducing cell death programs [19]. As illustrated by the selected genes shown in Figure 2, sustained gastrin treatment was required for increased expression of the main regulator of the UPR, *Hspa5* (*BiP*/*Grp78*), the transcription factor *Atf4* and the downstream mediators *Herpud1*, *Ddit3* (*Chop*) and *Chac1*[18-21]. Since HERPUD1 is reported to mediate cell protection via the stabilization of ER Ca^2+^ homeostasis [22], its activation indicates a possible mechanism for enhanced cell survival by sustained gastrin treatment. In contrast, the ATF4 target gene *Ddit3* (*Chop*), and DDIT3 target gene *Chac1* encode proteins associated with the pro-apoptotic effect of the UPR [21,23]. Our results demonstrate that sustained gastrin treatment is required for induction of several ER stress/UPR genes and that the proteins encoded by these genes are associated with both pro-survival and pro-apoptotic effects. Thus, it cannot be directly deduced from these results in which direction the subsequent cellular response will be affected.

**Figure 3:**
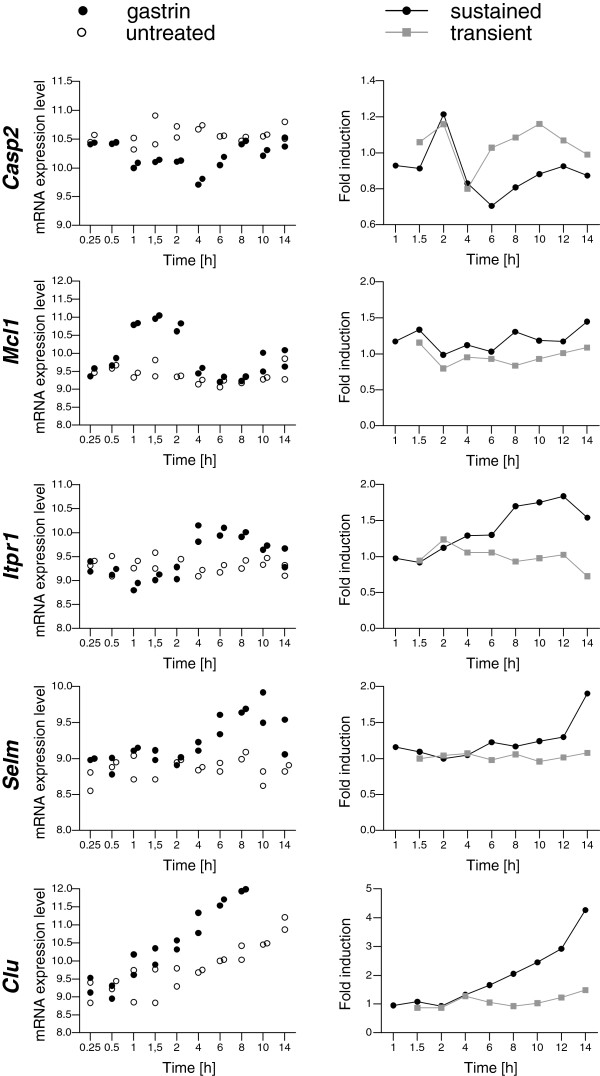
**Pro- and anti-apoptotic genes are differentially expressed in transiently and sustained gastrin treated cells.** The panels show gene expression time profiles for selected apoptosis-associated genes, differentially expressed in transiently *versus* sustained gastrin treated cells. Data from two independent microarray experiments are shown as described in the legend to Figure 2B. *Casp2*: caspase 2; *Mcl1*: myeloid cell leukemia sequence 1; *Itpr1*: inositol 1,4,5-trisphosphate receptor, type 1 (synonym: *Ip3r1*); *Selm*: selenoprotein M; *Clu*: clusterin.

The anti-apoptotic effect of gastrin is well documented [24-27], and it is therefore of interest to take a closer look at the relation to apoptosis among the genes differentially regulated in the transient *versus* sustained mode. The pro-apoptotic *Casp2* exhibits higher expression levels in the transient mode than in the sustained mode, while the anti-apoptotic genes *Mcl1*, *Itpr1*, *Selm* and *Clu* were more strongly increased by sustained gastrin treatment (Figure 3). We have previously shown that CLU is required for the anti-apoptotic effect of gastrin in our AR42J adenocarcinoma cell line model [24]. As a versatile stress-induced chaperone, CLU is suggested to be involved in protein homeostasis via unfolded protein and ER stress responses [28], and was recently shown to function cooperatively with GRP78 (Bip) to mediate anti-apoptotic effect in the mitochondria pathway [29]. MCL1, a member of the Bcl-2 protein family which inhibits release of cytochrome c from mitochondria [30], has previously been shown to be involved in the anti-apoptotic effect of gastrin in human adenocarcinoma cells (AGS-GR) stably transfected with the gastrin/CCK-2 receptor [31]. The ITPR1 receptor mediates Ca^2+^ release from ER and may be involved in mediating anti-apoptosis by interacting with anti-apoptotic members of the Bcl-2 family, including MCL1 [32], while SELM is shown to decrease Ca^2+^ release from ER in response to oxidative stress and reduce apoptotic cell death [33]. Taken together, our gene expression microarray analysis indicates that sustained but not transient gastrin treatment affects the balance between apoptosis and survival via several cross linked mechanisms including communication between ER and mitochondria which is critical for cellular decision making [29].

### Sustained gastrin treatment inhibits serum starvation-induced apoptosis via a PKC-dependent mechanism

**Figure 4:**
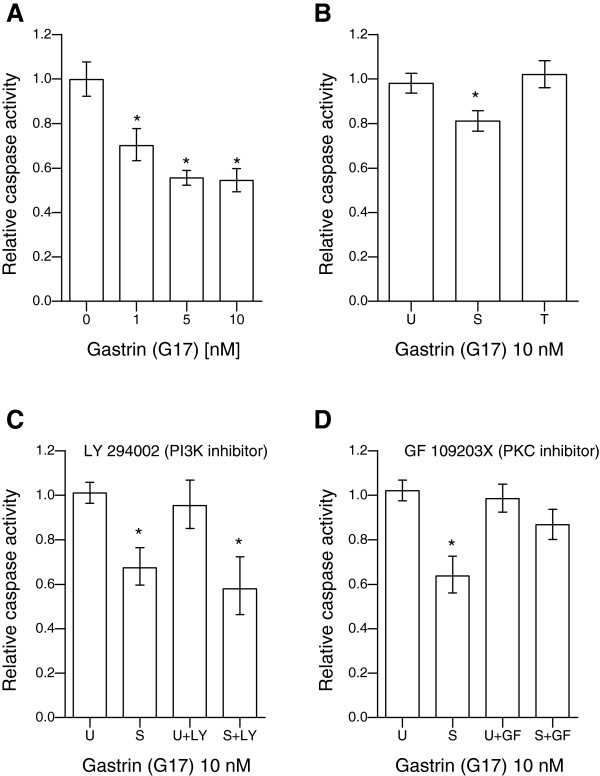
**Sustained gastrin treatment has an anti-apoptotic effect involving PKC-dependent mechanisms.** Apoptosis was induced in AR42J cells by serum starvation for 72 h and measured using Caspase Glo 3/7 assay. **A**: Caspase activity in cells treated with gastrin. **B**: Caspase activity in untreated (U), sustained (S) or transiently (T) gastrin treated cells. **C**-**D**: Caspase activity in cells pretreated with inhibitors of PI3K (LY) or PKC (GF) before cultivating in the absence (U) or presence of gastrin in a sustained mode (S). The data were normalized to the median intensity of untreated cells in each independent experiment, and is shown as mean relative caspase 3/7 activity of three independent experiments (6 technical replicates in each independent experiment). Error bars represent 95% CI. * Bonferroni-adjusted p-value < 0.05; significant difference from untreated cells with or without inhibitor.

To examine the effect of gastrin signalling duration on cell survival, we induced apoptosis in AR42J cells by serum starvation for 72 h and measured caspase activity in cells subjected to sustained or transient gastrin treatment. We found that sustained gastrin treatment significantly reduced effector caspase activity (Figure 4A), which is in accordance with our previous results using both TUNEL and caspase assay for detection of apoptotic cells [24]. This anti-apoptotic effect was not detected in cells treated with gastrin in a transient mode (Figure 4B). Thus, manifestation of the gastrin-induced anti-apoptotic effect which is well documented in literature [24-27], requires sustained gastrin treatment and is not induced when cells are exposed to gastrin for only 1 hour.

Since both the PI3K/AKT (PKB) pathway and ERK1/2 activation have been shown to be important in mediating the biological effects of gastrin, including anti-apoptosis [25,26], we next set out to determine the involvement of these two signalling pathways in our anti-apoptosis model system. Involvement of ERK1/2-signalling was assayed by the use of a PKC-inhibitor, since ERK1/2 activation has been shown to be mediated by PKC-Src/Ras/Raf/MEK/ERK1/2 cascade and/or by direct activation of Raf by PKC in the gastrin response [9]. The anti-apoptotic effect of sustained gastrin treatment was not influenced by the PI3K inhibitor (Figure 4C). In the presence of the PKC inhibitor gastrin still resulted in a small decrease in caspase 3/7 activity. However, this decrease was smaller than in the absence of the PKC-inhibitor, and the anti-apoptotic effect was no longer statistically significant (Figure 4D). We therefore conclude that PKC and its downstream effector ERK1/2 may play a decisive role in the sustained gastrin-induced anti-apoptotic effect. This observation complies with results reported by others showing that a specific inhibitor of the ERK1/2 activator MEK1 blocked gastrin-induced anti-apoptosis in AR42J cells [26].

### Genes that require sustained gastrin treatment for late upregulation include both primary and secondary responders

**Figure 5:**
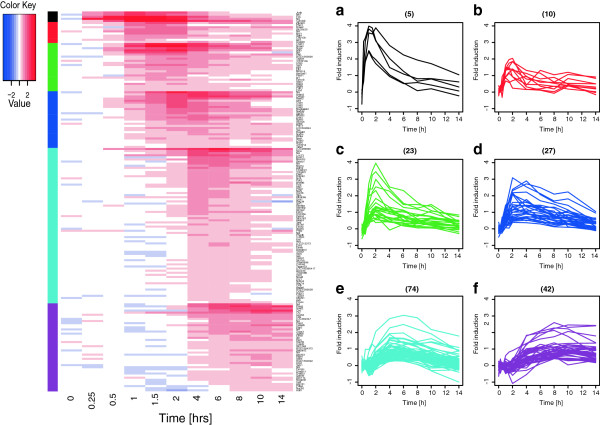
**Temporal expression profiles of early, delayed and late gastrin-induced genes.** A subset of 181 markedly gastrin-induced genes with differing expression patterns in transient *versus* sustained mode were used to further characterize the temporal profiles. The data were extracted from the independent time series microarray experiment where sustained gastrin treated cells were harvested at 10 different time points between 15 min and 14 h. The samples from untreated control cells were harvested at time zero and throughout the time course (11 time points). These genes were grouped by time profiles and peak expression (based on mean fold induction of sustained *versus* untreated cells at the same time point, N=2) into 6 groups as illustrated in the heat map and panel **a**-**f**. See details in the main text.

Cellular decision making in response to growth factors like gastrin is driven by transcriptional cascades involving primary response genes dominated by regulators of transcription and signal transduction and secondary response genes which depend on *de novo* protein synthesis and which include a high fraction of biological response effector genes [34,35]. To investigate primary and secondary transcriptional response mechanisms underlying the distinct biological responses to transient *versus* sustained gastrin signalling, we focused on genes that were markedly upregulated by gastrin and expressed at lower levels in the transient mode. This 181 gene subset can be grouped according to their time profiles as follows: 15 early genes peaking before 2 h (Figure 5 a-b); 50 delayed genes with peak expression at 2–4 h (Figure 5, c-d); and 116 late genes upregulated 4–14 h (Figure 5, e-f)(see Additional file 4: Table S4 for details). Genes dependent on *de novo* protein synthesis were identified by investigating the effect of the protein synthesis inhibitor CHX on gastrin-induced gene expression responses. This was done using data from a genome wide time series experiment where the cells were treated by gastrin in the absence and presence of CHX. To minimize misinterpretation due to the confounding effect of CHX [34,36,37], the temporal profiles (7 time points; 0–10 h) of treated and control cells were manually evaluated: For those genes where the gastrin response in the presence of CHX was higher than the response with CHX alone, the gene was classified as a primary gene not dependent on *de novo* protein synthesis for gastrin-induced expression. Gastrin induced genes that were not upregulated in the presence of CHX were classified as secondary genes, i.e., genes whose transcriptional activation depends on *de novo* protein synthesis of one or several factors that must be induced by gastrin previous to these secondary genes. In cases where the effect of gastrin could not be separated from the confounding effect of CHX, the gene was classified as uncertain. Among the subset of 181 genes upregulated in sustained treated cells and lower expressed in transiently treated cells, 115 were classified as primary genes, 48 as secondary and 18 as uncertain. The fraction of primary genes was successively reduced from 100% among the early genes to 40% among the late genes, while 43 of the 48 secondary genes were found among the late gastrin induced genes (Table 2 and Additional file 4: Table S4). Temporal expression data for genes in the subset are shown in Additional file 5.

**Table 2:** Classification of primary and secondary gastrin induced genes lower expressed in transiently treated cells

	**Primary genes**^**1 **^**(N=115)**						**Secondary genes**^**2 **^**(N=48)**					
Fraction												
Per cent (%) genes in each group:	100	90	70	74	65	40	0	10	9	7	30	50
**Temporal profiles**^**3**^	a	b	c	d	e	f	a	b	c	d	e	f
Molecular function ^4^ ( N=163)	5	9	16	20	48	17	0	1	2	2	22	21
Regulation of gene expression (N=22)	3	1	6	7	0	1	0	0	0	2	1	1
Transcription factors	3	0	3	5	0	0	0	0	0	0	0	1
Co-transcription factors	0	1	3	2	0	1	0	0	0	2	1	0
Signal transduction (N=44)	0	4	4	7	15	4	0	0	1	0	5	4
Receptors	0	0	0	2	1	1	0	0	0	0	1	0
Ligands	0	0	2	1	6	2	0	0	1	0	1	2
Intracellular signalling proteins	0	4	2	4	8	1	0	0	0	0	3	2
Transporters (N=15)	0	2	0	1	7	0	0	0	1	0	1	3
Channel and/or ER related	0	1	0	1	6	0	0	0	1	0	0	3
Other transporters	0	1	0	0	1	0	0	0	0	0	1	0
Protein Binding (N=23)	0	1	1	2	9	3	0	0	0	0	2	4
Chaperone activity	0	1	0	0	1	1	0	1	0	0	1	4
Other protein binding	0	0	1	2	8	2	0	0	0	0	1	0
Enzymes (N=39)	0	0	2	1	11	6	0	0	0	0	11	8
Proteases	0	0	2	0	4	1	0	0	0	0	0	6
Aminoacyl tRNA synthases	0	0	0	0	0	0	0	0	0	0	6	0
Other enzymes	0	0	0	1	7	5	0	0	0	0	5	2
Other functions (N=20)	2	1	3	2	6	3	0	0	0	0	2	1

^1^ Genes independent on *de novo* protein synthesis for gastrin induced expression (115 of 181 selected genes); ^2^ Genes dependent on *de novo* protein synthesis for gastrin induced expression (48 of 181 selected genes); number of uncertain genes: N= 18; ^3^ Temporal profiles are illustrated in Figure 5; ^4^Molecular functions are defined based on data from MetaCore tool of GeneGo package [15] Gene Ontology Annotation (UniProt-GOA) Database GOA [38] and literature.

Even though we found that the fractions of primary genes are reduced throughout the time course, most (~70%) of the genes in the subset were classified as primary genes that do not depend on *de novo* protein synthesis of upstream regulators (Table 2 and Additional file 4: Table S4). This finding is somewhat surprising, since previous reports have indicated that signal-responsive genes are induced in waves where the group of genes that requires *de novo* protein synthesis for expression and that is normally expressed in later waves, is far more numerous than the group of early primary response genes [39]. One explanation for our apparently conflicting observations implying that a large fraction of delayed and late genes are indeed primary genes, is that we recorded the genome-wide transcriptome at a high number of time points up to 14 h and in this way are able to display the gastrin-induced transcriptional cascades at high temporal resolution. This approach revealed that in addition to early genes transcriptionally activated within 2 h, also a high proportion of the delayed and late gastrin induced genes were upregulated independently of *de novo* protein synthesis. The observation that many delayed induced genes were primary genes are in accordance by results from a global time series gene expression analysis in human glioblastoma cells where Tullai *et al*. [40] showed that of the 133 genes induced within 4 h of human platelet-derived growth factor (PGDF) treatment, 49 were primary immediate early genes and 58 were primary delayed genes. Detailed analyses of the relation between temporal gene expression profiles beyond 4 h and dependence on *de novo* protein synthesis have to our knowledge not been reported. Thus, our present study contributes to shed light on these aspects of growth factor response transcriptional dynamics.

To further characterize the subset of primary and secondary gastrin induced genes that were lower expressed in the transient mode, we annotated their molecular function based on Gene Ontology (GO) and literature and grouped them into five main categories: regulation of gene expression, signal transduction, transporters, protein binding and enzymes. Regulators of gene expression are mainly found among the early and delayed genes, while genes encoding signal transduction proteins are spread throughout all temporal profile groups. Late genes are dominated by effectors involved in transport, enzyme and chaperone activity (see Table 2 and Additional file 4 for details).

The group of late genes upregulated only by sustained gastrin treatment comprises 65 primary and 43 secondary mRNA transcripts. Both primary and secondary late gene products are involved in transport and protein binding including chaperone activity. In addition, 35 late genes are classified as enzymes (Table 2 and Additional file 4: Table S4). Among these, enzymes are possible effector genes specifically associated with processes in the exocrine pancreas: The protease coding genes *Ctrc*, *Prss1*, *Prss3*, *Sec11c* and *Cela3b* are all highly expressed in the pancreatic acinar derived AR42J cell-line. Sustained but not transient gastrin treatment significantly enhanced the mRNA levels of these enzymes (Additional file 5). The genes were not induced in the presence of CHX and were therefore classified as secondary gastrin-induced genes. To our knowledge, gastrin has not previously been shown to activate gene expression of these proteases.

**Figure 6:**
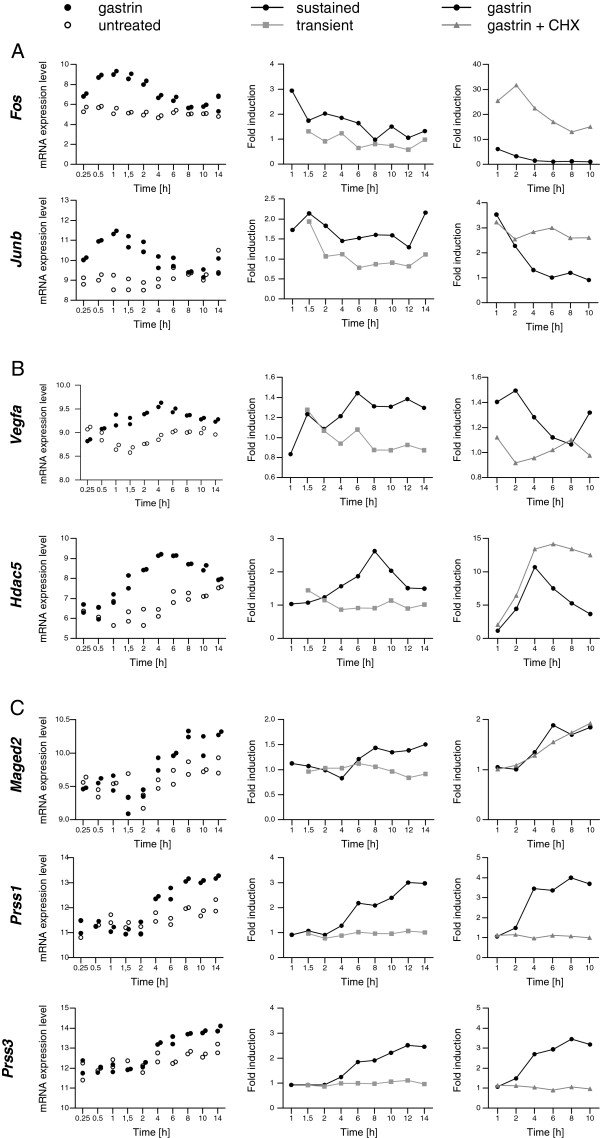
**Time profiles for selected early (A), delayed (B) and late (C) genes differentially expressed in transiently versus sustained gastrin treated cells.** The panels show data from three independent time series microarray experiments. Left panels: mRNA expression level (normalized log2-transformed signal intensities) for untreated (open dots) and sustained gastrin treated (black dots) cells. Experimental protocol is described in the legend to Figure 2B. Middle panels: Gastrin induced gene expression in transiently (grey lines) and sustained (black lines) treated cells. The data is shown as mean fold induction relative to untreated cells at the same time point (N=2; see Figure 1A for details). Right panels: The effect of sustained gastrin treatment was measured in the presence (grey lines) and absence (black lines) of cycloheximide (CHX). The data is shown as mean fold induction relative to either untreated cells (gastrin *versus* untreated) or relative to CHX treated cells (gastrin and CHX *versus* CHX) at the same time point (1-10 h). The early primary genes *c-Fos* and *Junb* as well as the delayed primary gene *Hdac5* are super-induced in the presence of CHX. Thus, these genes are probably repressed by other gastrin-induced repressors dependent on de novo protein synthesis [37]. The late gene *Maged2* is a primary gene. The delayed gene *Vegfa* and the late genes *Prss1* and *Prss3 (LOC362347)* are secondary. Hdac5 is a co-transcription factor involved in histone modification and shown to control cell-cycle progression and survival of human cancer cells [45]. VEGFA acts on endothelial cells and has various effects, including mediating increased vascular permeability, inducing angiogenesis and cell growth, promoting cell migration, and inhibiting apoptosis [46]. Maged2 has been classified as a co-transcription factor and are found elevated in e.g., goblet cell adenocarcinoids compared to normal mucosa [47]. Prss1 and Prss3 are discussed in the main text.

Proteases can contribute to cancer development by several mechanisms [41]. For example, in human pancreatic cancer, PRSS3 upregulates the ligand VEGFA via the proteinase-activated receptor 1 (PAR1)-mediated ERK1/2 pathway; and blockade of PRSS3-triggered ERK signalling is shown to delay the progression of metastasis and prolong the survival of mice bearing PRSS3-positive human pancreatic tumours [42]. Others have shown that gastrin enhances VEGFA gene expression in human colon cancer cells stably transfected with a wild-type CCK2 receptor [43]. Moreover, somatic mutations that increase gastrin/CCK2 receptor activity increase the secretion of VEGF and promote cell migration and angiogenesis in colorectal and gastric cancer cells [44]. Our findings that upregulation of both *Prss3* and *Vegfa* require sustained gastrin signalling indicate that the proteins encoded by these genes may be involved in hypergastrinemia associated pathophysiological processes (see Figure 6 and discussion below).

Overall, scrutiny of the 181 selected temporal gene expression profiles revealed that the differences in transiently *versus* sustained treated cells were subtle for early response genes and more substantial for delayed or late gastrin induced genes; and genes that are only upregulated in the sustained mode comprise both primary and secondary genes (see Additional files 4 and 5 for extensive analysis). Exemplified time profiles for primary and secondary genes are shown in Figure 6. Primary early genes like c-*Fos* and *Junb* were upregulated in both sustained and transiently gastrin treated cells. However, their mRNA levels returned to baseline earlier in cells treated in a transient *versus* a sustained mode (Figure 6A). Delayed gastrin-responsive genes like primary *Hdac5* and secondary *Vegfa* were also temporarily upregulated in the transient mode compared to unstimulated control at early time points, but only sustained gastrin treatment induced high and prolonged expression (Figure 6B). Interestingly, both late primary genes like *Maged2* and late secondary genes like *Prss1* and *Prss3* were only detected in cells treated in a sustained mode (Figure 6C). This suggests that gastrin directs duration-dependent gene expression via two different routes: *i*) via signal transduction mechanisms that directly trigger transcriptional activation of primary genes throughout both early and late stages of the time course and *ii*) via mechanisms that depend on increased expression of transcriptional regulators responsible for upregulation of the late secondary genes.

### Sustained gastrin treatment is required for extended ERK1/2 activation

**Figure 7:**
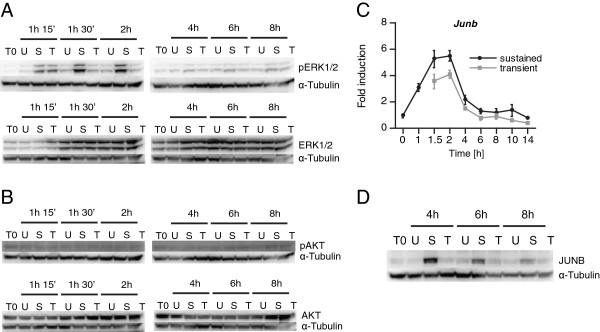
**Prolonged activation of ERK1/2 and expression of JUNB are dependent on sustained gastrin treatment.** Sustained and transiently gastrin treated cells were grown and harvested as described in Material and Methods. **A**-**B**: Activation of ERK (**A**) and AKT (**B**) were analysed at the indicated time points; starting 15 min after gastrin was removed in the transient protocol. Western Blot images of phospho-ERK1/2, total ERK1/2, phospho-AKT and total AKT in untreated (U), sustained (S) and transiently (T) gastrin treated cells. T0: time point zero. Results show one representative of three independent experiments. **C**: The duration and magnitude of mRNA expression of the AP-1 component *Junb* were measured by qRT-PCR analysis in cells treated by gastrin in a sustained or transient mode relative to untreated controls at time point zero in one representative experiment (mean fold induction +/− SD of three technical replicates). **D**: Western Blot image of JUNB protein in whole cell lysate at T0 and 4, 6 and 8 h of untreated (U), sustained (S) and transiently (T) treated cells. Result show one representative of three independent experiments.

Since our data indicated that a large fraction of gastrin induced genes differentially expressed in transiently *versus* sustained treated cells are regulated by post-translational direct signalling mechanisms, it was of interest to examine the effect of the duration of gastrin treatment on signalling pathways known to be activated by the gastrin/CCK2 receptor [9]. We therefore analysed AKT and ERK1/2 phosphorylation in cells treated with gastrin in a transient or a sustained mode; starting at 75 minutes, i.e. 15 min after gastrin was removed in the transient protocol. Our results show that whereas ERK1/2 activity remains elevated for several hours in cells treated in a sustained mode, the level of phosphorylated ERK1/2 returned to base-line within 60 min after gastrin was removed in transiently treated cells (Figure 7A). The level of phosphorylated AKT was low in both untreated and gastrin treated cells in this time period (Figure 7B), which is in accordance with finding by others showing that gastrin induced AKT phosphorylation peaks 5–30 min after treatment in AR42J cells [25] (i.e., in a time interval before the 75 minutes start of our observations). Thus, while AKT phosphorylation is not prolonged by sustained gastrin treatment, ERK1/2 phosphorylation, which is known to involve upstream PKC [9], is strongly elevated at later time points in sustained but not transiently treated cells.

It is well known that the duration of ERK1/2 activation is involved in directing cellular outcomes. For example, nerve growth factor (NGF) and heregulin (HRG) induce prolonged phosphorylation (i.e., activation) of ERK1/2 and subsequent differentiation in PC-12 and MCF-7 cells, respectively. Epidermal growth factor (EGF), on the other hand, induces transient phosphorylation of ERK1/2 and proliferation in the same cells [6,48-52]. Similarly, our findings that sustained gastrin treatment is required for the sustained activation of the PKC /ERK1/2-pathway as well as for PKC-dependent activation of anti-apoptosis (Figure 4), indicate that the duration of ERK1/2 activation translates duration of gastrin signalling into pro-survival cell fate in the AR42J adenocarcinoma cell model system.

The magnitude of ERK activity can be regulated by scaffold proteins [49]. We have recently shown that the gastrin induced MAPK scaffold protein MEK partner 1 (MP1) is important for gastrin induced phosphorylation of ERK1 and ERK2 in the human gastric adenocarcinoma cell line AGS-G_R_[53]. Qualitative and quantitative differences in ERK activity can also be regulated at the receptor level [49]. For example, the EGF receptor has been reported to undergo rapid internalization and degradation upon EGF stimulation with subsequent transient ERK activation [49]. Less is known about how prolonged gastrin stimulation affect the CCK2 (gastrin) receptor. However in our genome-wide time series experiments we find that gastrin induces gene expression of CCK2R and that the induction of *Cck2r* mRNA is somewhat higher in sustained *versus* transiently treated cells. The *Cck2r* was highly expressed in the presence of CHX which indicates that *Cck2r* is a primary gastrin-induced gene and negatively feedback-regulated at the mRNA level (data not shown). This is in accordance with findings by others showing that gastrin increases CCK2R expression at the mRNA and protein levels, both in cell cultures [54,55] and *in vivo*[55]. More investigation is needed to address the status of the gastrin receptor upon different duration of gastrin treatment. However, our finding that sustained gastrin treatment induces prolonged ERK1/2 activation suggests that the gastrin receptor desensitizes relatively slowly compared to the EGF receptor.

### Molecular responses dependent on sustained gastrin treatment involve AP-1

Sustained MEK/ERK activation is known to enhance the activity of several members of the JUN- and FOS-family of proteins [3,48] which are involved in transcription regulation through homo- or heterodimeric AP-1 complexes that can also involve proteins from the closely related CREB/ATF- and Maf-families [56]. Thus, it was of interest to examine the potential involvement of AP-1 in gastrin-mediated transcriptional activation of the primary and secondary genes described in Figure 5 and Table 2. We found that binding sites for AP1, ATF and CREB were overrepresented (Bonferroni corrected *p* value≤0.05) among primary upregulated genes (Additional file 6: Table S5). None of these transcription factor binding sites were overrepresented among the secondary genes. Interestingly, binding sites for AP-1 were overrepresented also among primary genes with late expression profiles (d-f, Figure 5). These findings indicate that AP-1 transcription factors may play a central role in gastrin induced gene expression that requires sustained treatment and is mediated via post translational signalling events independent of *de novo* protein synthesis.

Detailed analysis of JUNB mRNA and protein showed that although the difference at mRNA level was subtle (Figure 7C), protein levels were substantially higher and more prolonged in cells treated in the sustained mode (Figure 7D). A potential mechanism underlying this observation may be stabilization of JUNB by phosphorylation due to prevention of its degradation via proteasome pathways. Such inhibition of proteasome degradation by sustained ERK1/2 activation has been reported in other model systems [3,48]. Our results suggest that enhanced JUNB protein levels downstream of ERK1/2 signalling may play a decisive role in differential gene expression responses to sustained *versus* transient gastrin treatment.

**Figure 8:**
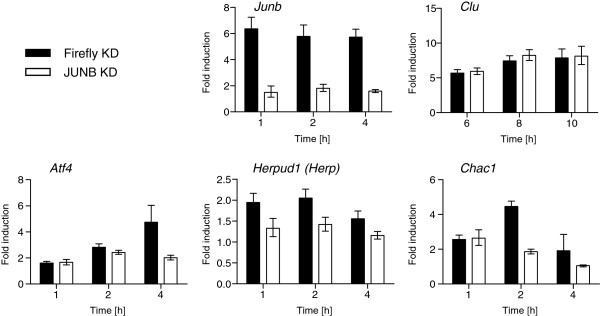
**JUNB is involved in sustained gastrin induced expression of ER stress/UPR related genes.** Cells with JUNB knocked down (JUNB KD) and control cells (Firefly KD) were harvested at indicated time points after sustained gastrin treatment, and mRNA expression level for selected genes were measured by qRT-PCR analysis. Results show fold induction of gastrin treated cells relative to untreated control cells (mean +/− SD of three technical replicates). The mRNA expression level of *Atf4* was 57% lower in JUNB KD cells compared to control cells at 4 h. *Herpud1* mRNA levels were ~30% lower in JUNB KD cells at 1, 2 and 4 h, while *Chac1* was repressed 58% and 45% at 2 and 4 h, respectively when JUNB was knocked down. Figures represent one of two independent experiments.

To further examine the role of JUNB, we studied gastrin-induced gene expression in cells where JUNB had been knocked down by retrovirus-based shRNA (JUNB KD). The efficiency of the RNAi was demonstrated by the fact that *Junb* mRNA was suppressed 70% in JUNB KD compared to control cells (Firefly KD) (Figure 8). By focusing our analysis on UPR related genes differentially expressed in transiently *versus* sustained gastrin treated cells (Figure 2A), we found that JUNB knock down suppressed gastrin induced transcriptional activation of the transcription factor *Atf4*, involved in the PERK-eIF2α pathway of the UPR, and the downstream mediator genes *Herpud1* and *Chac1*. Gastrin-induced *Clu* mRNA levels were similar in JUNB KD and control cells indicating that JUNB is not critically involved in the regulation of this gene (Figure 8).

These results suggest that JUNB is involved in sustained gastrin induced expression of *Atf4* which may subsequently affect downstream ATF4 mediated gene expression, thus contributing to the differences seen between sustained and transient gastrin treatment. JUNB can regulate gene transcription both positively and negatively as partners in AP-1 dimers [57,58]. The stress induced ATF4 transcription factor has numerous dimerization partners including members of AP-1 and C/EBP family of proteins [59]. In addition to *Herpud1* and *Chac1*, ATF4 is known to mediate upregulation of several of the sustained gastrin regulated genes identified in this work, e.g. *Atf3*, *Mcl1* and *Vegfa*[59,60]. Recently, JUNB was found to inhibit stress-induced apoptosis [61], and ATF3 has been identified as a downstream target of JUNB in the survival mechanism [62]. Taken together, our results indicate that the distinct mRNA expression patterns in cells treated in transient and sustained modes are, at least in part, linked to the duration of ERK1/2 activation and expression of the AP-1 components like JUNB as well as the ATF subfamily proteins ATF4 and ATF3 for which the mRNA expression levels are prolonged only in the sustained mode.

## Conclusions

Genome-wide time series analyses are an important approach to capture the dynamics of stimuli-induced gene expression responses over time [63]. The major contribution of this study is that we by extensive time series gene expression analysis and molecular studies characterize the distinct effects of duration of gastrin treatment on intracellular signalling events and gene expression in adenocarcinoma cells. We further show that the differences in global gene expression were reflected in distinct cellular responses in transiently and sustained gastrin treated cells since our results point to the possibility that a sustained high level of gastrin may affect the survival/apoptosis balance. Our findings may contribute to a better understanding of how gastrin-mediated regulation of gene expression relates to pathophysiological processes in response to high and sustained levels of gastrin. Although we have limited our investigation to one cell line in the present study, our findings provide interesting new hypotheses for further studies *in vivo* as well as other model systems.

## Methods

### Cells and reagents

The AR42J cells (rat pancreatic acinar cell derived, ATCC, Rockville, MD) were cultured at 37°C in humidified 5% CO_2_ Dulbecco’s modified Eagle’s medium (DMEM) containing 4.5 g/l glucose supplemented with 1% (v/v) penicillin-streptomycin, 0.1 mg/ml L-glutamine, 1 μg/ml fungizone, 1mM sodium pyruvate (all Gibco, Invitrogen), and 15% fetal bovine serum (FBS, Lonza BioWhittaker, Basel, Switzerland). Gastrin-17 (G-17) was purchased from Sigma-Aldrich (St. Louis, MO). LY 294002 and GF 109203× were obtained from Calbiochem (La Jolla, CA).

### Time-series experiments in AR42J cells

The rat pancreatic acinar cell derived cell line AR42J has been used as a model system to study exocrine pancreas [64]. Furthermore, AR42J cells express gastrin receptors endogenously and can therefore be used as a model system to study gastrin responses like proliferation [65], differentiation [66] and apoptosis [24-27]. In the time-series experiment we treated cells with 10 nM gastrin in accordance with several other studies investigating gastrin-induced responses in the AR42J cell line [25,54,67]. The cells were grown in 6-well plates (3 ×10^5^ cells/well) for 72 h. Then the growth medium was replenished with 2 ml serum-free DMEM, and the cells serum starved for 20–24 h before adding gastrin. Treated and untreated cells were grown in parallel and harvested (pool of 2–3 technical replicates) at several time points, as indicated in the figures. In experiments with the protein synthesis inhibitor cycloheximide (CHX), pre-treatments with CHX (10 μg/ml) were initiated 30 min before gastrin was added. In experiments with transient *versus* sustained gastrin treatment, the growth medium of untreated and gastrin treated cells was removed 1 h after gastrin treatment; the cells were then washed with serum-free medium before fresh serum-free medium with gastrin (sustained gastrin treated cells) or without gastrin (transiently gastrin treated or untreated cells) was added. The cells were harvested for RNA isolation or protein lysate as described below.

### RNA isolation

Total RNA from AR42J cells and frozen oxyntic samples was isolated using RNeasy Mini kit (Qiagen, Germantown, MD) according to the manufacturer’s instructions. RNA quantity and purity was assessed using a Nanodrop spectrophotometer (Nanodrop Technologies, Rockland, DE), and RNA integrity controlled by measuring RIN values using a Bioanalyser capillary gel electrophoresis assay (Agilent Technologies, Palo Alto, CA). The RNA samples were kept at −80°C until further processing.

### Genome-wide gene expression analysis on Illumina ExpressionBeadChips

RNA amplifications and hybridization were performed at the NTNU Genomics Core Facilely (GCF). Briefly, RNA was amplified with Ambion's Illumina® TotalPrep RNA Amplification kit (cat no AMIL1791) using 400 ng of total RNA as input material. The *in vitro* transcription (IVT) amplification that incorporated biotin-labeled nucleotides was performed overnight (14 hours) at 37°C. After the amplifications the cRNA concentrations where checked with NanoDrop ND-1000 and cRNA quality was controlled by BioRad’s Experion electrophoresis station. A total of 750 ng of each biotin-labeled cRNA sample was hybridized to Illumina’s RaRef-12-v1 Expression BeadChips at 58°C overnight (17 h) according to the manufacturer’s instructions [68]. The hybridized biotinylated cRNA was detected with 1ug/ml Cyanine3-streptavidine, (GE Healthcare Biosciences) and the Beadchips were scanned with Illumina BeadArray Reader (Factor=1, PMT=521, Filter=100%). Numerical results were extracted with Bead Studio v3.0.19.0 without any normalization or background subtraction.

Data of gastrin-regulated gene expression which compared treatment in the transient (1 h) *versus* sustained (14 h) mode is a time course reference design with a non-treated reference measured at each sampled time point. The data was normalised by *loess* adjustment within time points and average quantile normalised between time points. The data was analysed using the *Limma* (ver. 3.12.1) Bioconductor package [69]. Replicated treated and untreated (reference) samples were compared using two separate linear models. The first model covers each time point from 0–4 h and the second and main model encompasses the remaining samples (6–14 h). Both models were tested for overall significance across time using a moderated F-test. Genes with a FDR adjusted p-value < 0.05 were taken as significant. The ratio between treated and untreated (reference) sample at each time point was averaged between biological replicates, and 403 significant genes were visualised in a heat map (Figure 1B) where genes (rows) are ordered by hierarchical clustering using Euclidean distance and Ward agglomeration. Mapping between Illumina probe identifiers and gene symbol was fetched from official Illumina annotation files (RatRef-12_V1_0_R5_11222119).

### Database submission of microarray data

In addition to the time series experiment comparing treatment in transient (1 h) *versus* sustained (14 h) mode, we used data from two other time series experiments to analyse temporal time profiles of early and late gastrin responsive genes and assess gene expression in the presence and absence of the protein synthesis inhibitor cyclohexamide (CHX) to distinguish primary and secondary genes. The microarray data were prepared according to minimum information about a microarray experiment (MIAME) recommendations [70] and deposited in the Array Express [71]. Detailed information about the microarray designs and raw data files from the experiments are accessible by use of these accession numbers: GSE32869, and E-MTAB-1268 (Illumina platform).

### Enrichment analysis of pathways, GO-annotations and transcription factor binding site (TFBS)

Canonical pathways and networks most significantly associated by the genes differentially expressed in transienly *versus* sustained gastrin stimulated cells were determined by GeneGo MetaCore^TM^[15] enrichment analysis with the general p-value threshold p<0.05 for the data inputs. Molecular functions were defined based on data from MetaCore tool of GeneGo package i.e., Network objects (object types), Gene Ontology Annotation (UniProt-GOA) Database GOA [38] and literature. Transcription factor binding site (TFBS) enrichment was assessed for human orthologues of the rat genes by using DAVID, Database for Annotation, Visualization and Integrated Discovery, version 6.7 [72,73].

### Real time qRT-PCR

cDNA synthesis was performed using Transcriptor First Strand cDNA Synthesis Kit from Roche Diagnostics GmbH (Mannheim, Germany), as described earlier [24]. After the cDNA synthesis reaction, the samples were diluted 1:3 in sterile water. Quantitative RT-PCR (qRT-PCR) was performed using StepOnePlus, Real-Time PCR System from Applied biosystems and B-R SYBR Green SuperMix for iQ (Quanta Bioscience, Gaithersburg, MD). Each reaction contained SYBR Green SuperMix (2×) (12.5 μl), sense primer (2.5 μl of 3 μM), antisense primer (2.5 μl of 3 μM), cDNA template (2.5 μl), and sterile water (5.0 μl). Quantitative PCR thermal cycling program: 40 thermal cycles of 15 s at 95°C, 30 s at 60°C, and 40 s at 72°C. A dissociation curve was made to confirm primer specificity. Primers: (Rattus Norvegicus): *Clu*, sense: 5′-GCTCCATAGCCCAGCTTTAC-3′, antisense: 5′-ACTTCTCACACTGGCCCTTC-3′ [24]; *B2m*: sense: 5′-CGAGACCGATGTATATGCTTGC-3′, antisense: 5′-GTCCAGATGATTCAGAGCTCCA-3′ [24]; *Atf4*, sense: 5′-GTTGGTCAGTGCCTCAGACA-3′, antisense: 5′-CATTCGAAACAGAGCATCGA-3′ [74]; *Junb*, sense: 5′-AGCTAGCCTCCACGGAACT-3′, antisense: 5′-CTCCTGCTCCTCGGTGAC-3′; *Herpud1*, sense: 5′-TTGCACCTCGTGTGCAATGTGAGG-3′, antisense: 5′-ACTAGTGTTGTCCGGCTGCTCTGT-3′; *Chac1*, sense: 5′-CCTTCCACAGGGGCAGCGATAAGAT-3′, antisense: 5′-AACCTGGTATGCCACACCCCAAGTG-3′. Primers for *Junb*, *Herpud1* and *Chac1* were designed using Clone Manager 9 (Scientific & Educational Software, Cary, NC) or Primer-Blast [75]. All samples were run in triplicates, and relative mRNA expression levels were quantified using the ΔΔCt-method [76] with *B2m* as reference gene.

### Western blot analysis

Cells were washed twice in PBS, and harvested in 400 μl cold RIPA buffer (Thermo Scientific, Rockford, IL) containing 8 μl Halt™ Protease Inhibitor Single-Use Cocktail (100×) (Thermo Scientific) and 4 μl Halt™ Phosphatase Inhibitor Single-Use Cocktail (100×) (Thermo Scientific) per well. The lysate was homogenized using a syringe and needle (21 G), and cell debris removed by centrifugation (15 min, 14000 ×g, 4°C). The supernatant was stored at −80°C until further processing. The protein concentrations were measured using the Pierce® BCA Protein Assay Kit (Thermo Scientific) according to the manufacturer’s instructions. Gel electrophoresis and blotting were performed using the NuPAGE system from Invitrogen according to the manufacturer’s instructions; as previously described [24]. The membranes were blocked in TBST (50 mM Tris·HCl pH 7.5, 150 mM NaCl, 0.1% Tween 20) with 5% bovine serum albumin (BSA) (Sigma-Aldrich) for 1 h at room temperature, incubated with the primary antibody diluted in TBST with 1% BSA overnight at 4°C, and incubated with the secondary HRP-conjugated antibody diluted in TBST with 1% BSA for 2 h at room temperature. For detection of α-tubulin (loading control), blocking, washing, and antibody incubation were performed with SNAP i.d. Protein Detection System (Millipore, Billerica, MA) according to the manufacturer’s instructions, using TBST with 1% BSA for blocking and antibody dilution. Binding of secondary antibodies was visualized by use of the SuperSignal West Femto Maximum Sensitive Substrate (Thermo Scientific) and Kodak Image Station 2000R (Kodak, Pittsburgh, PA).

The following antibodies were used: monoclonal (mouse) anti-human α-Tubulin (reacts with mouse, rat and human) (1:300; sc-5286, Santa Cruz Biotechnology), polyclonal horseradish peroxidase (HRP) conjugated (goat) anti-mouse (1:3000; PO447, DAKO), polyclonal (rabbit) phospho-p44/42 MAPK (Erk1/2) (Thr202/Tyr204) (1:1000; #9101, Cell Signaling), polyclonal (rabbit) p44/42 MAPK (Erk1/2) (1:1000; #9102, Cell Signaling), monoclonal (rabbit) JunB (1:1000; #3753 Cell Signaling), polyclonal (rabbit) phospho-AKT (Ser473) (1:1000; #9271, Cell Signaling) and polyclonal (rabbit) AKT (1:1000;#9272, Cell Signaling), HRP-conjugated goat anti-rabbit (1:1000; #7074, Cell Signaling).

### Stable knockdown (KD) of JUNB by retrovirus-based RNAi

Eight 97-mer shRNA oligoes were designed to target Junb using the algorithm from [77]. The oligoes were cloned into MSCV P2Gm FF retroviral transfer vector (Addgene) using *Xho*I and *Eco*RI restriction enzymes (New England Biolabs). Correct clones were verified by sequencing. Packaged virus was obtained by cotransfection of 293FT cells (ATCC) with 1μg MSCV P2Gm FF transfer vector (Addgene), 0.5 μg MLV gag-pol (Addgene), and 0.5 μg CMV-VSVg (Addgene) expression vectors using Fugene6 (Roche) according to manufacturer’s protocol and as previously described [78]. Medium was replaced 24 h and 48 h after transfection, 293FT cell conditioned medium was collected, filtered through a 0.45μm filter, and applied to AR42J cells with 4 μg/ml polybrene (Sigma) [78]. Infected AR42J cells were GFP positive and selected with 2 μg/ml puromycin (Sigma). The cell lines with most efficient knock down were chosen for further analysis.

Hairpin sequence: shJunb

TGCTGTTGACAGTGAGCGAGCACTTCGTGTCTAAAGTCTATAGTGAAGCCACAGATGTATAGACTTTAGACACGAAGTGCGTGCCTACTGCCTCGGA

### Caspase assay

AR42J cells (1.5 × 10^4^) were seeded in white-walled 96-well plates (Perkin Elmer). The cells were incubated for one day before the growth medium was replenished with 150 μl serum-free DMEM with or without different doses of gastrin (1–10 nM). After 1 h, the medium was again replenished and gastrin added to cells treated in the sustained mode. Cells were further incubated for 72 h to induce apoptosis. Chemical inhibitors were added 30 min before the gastrin treatment. Caspase activity was measured using the Caspase-Glo 3/7 assay from Promega (Madison, WI) according to the manufacturer’s descriptions. Luminescence was measured using Wallac 1420 Victor3^TM^ plate reader (Perkin Elmer). All the data were log-transformed before statistical analysis. For presentation, the data were transformed back to the original scale and plotted as means with 95% confidence interval (CI) as error bars. Student's t-test was used to evaluate statistical significance, and we performed a Bonferroni correction for multiple testing at 0.05 level in each analysis.

## Competing interests

The authors declare that they have no competing interests.

## Authors’ informations

Linn-Karina M Selvik and Christina S. Fjeldbo should be regarded as joint First Authors.

## Authors’ contributions

LKMS carried out cell and molecular studies and helped to draft the manuscript. CSF participated in design and interpretation of microarray experiments, carried out cell and molecular studies and helped to draft the manuscript. AF participated in design of microarray experiments and performed statistical analysis and bioinformatics. TSS carried out cell and molecular studies and participated in interpretation of microarray experiments. KM carried out cell and molecular studies and participated in interpretation of microarray experiments. EA participated in design of microarray experiments and performed statistical analysis and bioinformatics. BD participated in design and analysis of microarray experiments and carried out initial microarray experiments. ML participated in design and statistical analysis of initial microarray experiments. ST carried out cell and molecular studies and participated in enrichment analysis. VB participated in design and analysis of microarray experiments and participated in enrichment analysis. AL raised funding, participated in study design and coordination, participated in interpretation of results, and helped to draft the manuscript. LT raised funding, participated in study design and coordination, participated in interpretation of results, and helped to draft the manuscript. TB participated in study design and coordination, participated in design, experimental work, analysis and interpretation of microarray experiments, carried out cell and molecular studies and drafted the manuscript. All authors read and approved the final manuscript.

## Supplementary Material

Additional file 1: Table S1 The file contains information about 403 genes with significantly different mRNA expression levels (adjusted p<=0.05) in cells treated with gastrin in a transient *versus* sustained mode (Accession number GSE32869). Click here for file

Additional file 2 **The file includes results from an initial cDNA microarray time series experiment (Accession number: E-MTAB-123). Figure S1A:** Schematic representation of stimulation protocol. **Figure S1B:** Graphical representation of experimental design of the two-colour cDNA microarray hybridizations. **Figure S2:** Number of differentially expressed genes. **Figure S3:** Analysis of microarray time series by dimension reduction methods. Click here for file

Additional file 3: Table S2 Enrichment analysis of 259 genes significantly lower expressed in transiently *versus* sustained gastrin treated cells. **Table S3:** Enrichment analysis of 144 genes significantly higher expressed in transiently *versus* sustained gastrin treated cells. All 403 genes are shown in the heat map in Figure 1B and Additional file 1. Click here for file

Additional file 4: Table S4 The file lists a subset of 181 gastrin-induced probes (177 unique IDs) with differing expression patterns in transient *versus* sustained mode which were used to further characterize the temporal profiles including information about gene expression in the presence and absence of a protein synthesis inhibitor (CHX) as well as molecular functions. The data are extracted from independent time series experiments in Accession numbers GSE32869 and E-MTAB-1268. Click here for file

Additional file 5 **Temporal profiles of gastrin-induced genes with differing expression patterns in transient *versus *sustained mode described in Figure** 5**and Additional file****4****: Table S4.** Time profiles as log2 expression data from three independent time series experiments are shown for each individual gene. Upper panels: transiently or sustained treated cells; Middle panels: sustained treated or untreated control cells. Lower panels: gene expression in the presence and absence of the protein synthesis inhibitor CHX. Click here for file

Additional file 6: Table S5 Identification of overrepresented transcription factor binding sites (TFBS) among the subset of upregulated genes lower expressed in transiently gastrin treated cells (described in Figure 5 and Additional file 4: Table S4). TFBS enrichment was assessed for human orthologues of the rat genes by using DAVID version 6.7 (Bonferroni corrected p-values). Click here for file
