# Supplementary material for: The duration of gastrin treatment affects global gene expression and molecular responses involved in ER stress and anti-apoptosis
Source: BMC Genomics. 2013 Jun 28;14:429. doi: 10.1186/1471-2164-14-429 (PMC3698217; doi:10.1186/1471-2164-14-429)
Supplement: Additional file 2 — The file includes results from an initial cDNA microarray time series experiment (Accession number: E-MTAB-123). Figure S1A: Schematic representation of stimulation protocol. Figure S1B: Graphical representation of experimental design of the two-colour cDNA microarray hybridizations. Figure S2: Number of differentially expressed genes. Figure S3: Analysis of microarray time series by dimension reduction methods. [file 1471-2164-14-429-S2.pdf]

ADDITIONAL FILE 2: additional results and discussion

To study molecular mechanisms involved in the gastrin response we initially performed a time series experiment in the AR42J cell line including treatment in both a sustained mode (24 h) as well as in transient mode (2 h). In order to be able to control for changes in gene expression levels in untreated cells during the 24 h observation period, we also sampled untreated control cells at all time points (Figure S1A). A total of three biological replicates were analysed to yield a data set based on 156 cDNA microarray hybridizations (Accession number: E-MATAB-123).

A

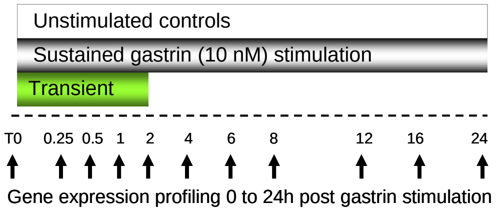

B

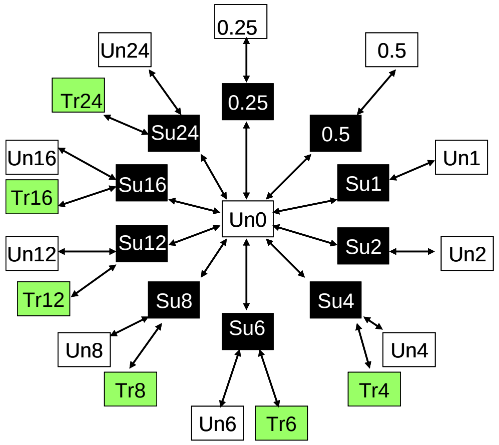

Figure S1A: Schematic representation of stimulation protocol. The AR42J cells were sub cultured in tissue culture flasks and serum starved for 24 h before medium was removed and replaced with serum-free medium containing 10 nM gastrin. Cells treated with gastrin in the sustained mode were harvested at 10 different time points between 15 min and 24 h. Cells treated with gastrin in the transient mode (gastrin containing medium was removed after 2 h and replaced with medium without gastrin) were harvested at 6 different time points between 4 and 24 h. The samples from unstimulated control cells were harvested at T0 and throughout the time course (11 time points).

Figure S1B: Graphical representation of experimental design of the two-colour cDNA microarray hybridizations. The structure of the graph determines which effect can be estimated and the precision of the estimate (see text above for details). The samples were hybridized to rat 15k cDNA microarrays with probes printed in duplicates. Dye-swap was performed for all conditions. The time course experiment was performed with three biological replicates (total of 156 hybridizations). In order to minimize bias connected to the day, person or order of hybridization, the hybridizations were randomized so samples from the three different biological replicates and the different technical replicates appeared randomly in the working list. Su: Sustained gastrin treatment; Tr: transient gastrin treatment; Un: Unstimulated control cells.

Differentially expressed genes during the gastrin response were determined at the 5% false discovery rate (FDR) level by using a linear model with a modified T-test after pre-processing by our optimized protocol [1]. The number of differentially expressed genes varied throughout the time course with an increasing number of differentially genes as the gastrin response progresses from 15 min throughout to 4 h, followed by a decline in number of differentially expressed genes over the remaining time period, as shown in Figure S2. A total of 754 genes were differentially expressed at 4 hours gastrin treatment compared to untreated cells at the onset of the experiment (T0). Of these,

443 genes were identified as up-regulated and 311 genes as down-regulated. Interestingly, 454 genes were differentially expressed in cells with sustained gastrin treatment compared to cells with transient treatment (at 4 h, 213 genes with higher and 241 genes with lower levels). Thus, the duration of gastrin stimulation substantially influences the gene expression levels. It is important to note that even in untreated cells a substantial number of genes changed their expression levels throughout the 24 h time course.

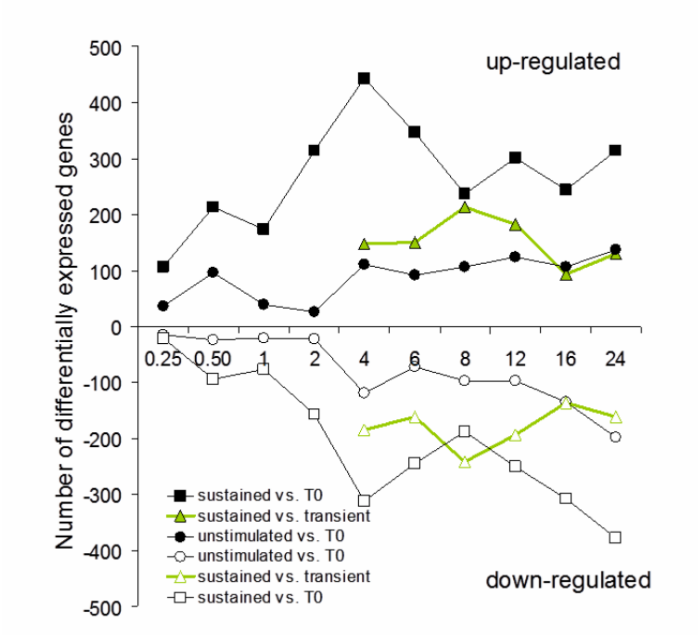

Figure S2: The figure shows the number of differentially expressed genes at the 5% FDR in sustained gastrin-treated cells and unstimulated control cells at the indicated time points compared to T0. The number of genes that were differentially expressed between cells treated with gastrin in the sustained mode compared to cells treated in the transient mode at corresponding time points are shown for 4 to 24 h. Solid points: up-regulated genes; open points: down-regulated genes. Only genes differentially expressed at 2 or more time points are included in the figure.

Microarray time series experiments are often analysed by unsupervised or explorative methods such as clustering, where genes are grouped together without defining explicit comparisons between groups of samples or tests for differential expression (for review, see [2]). Dimension reduction methods represent alternative analyses that do not include explicit comparisons between groups. These methods look for a few independent, or orthogonal, linear combinations of the original variables (i.e. genes) that can summarize interesting patterns of variability in a dataset. Comparison of gene expression patterns in transient *versus* sustained gastrin treated cells by dimension reduction methods [3] revealed that the gene transcript levels returned to baseline faster in cells subjected to transient treatment as shown in Figure S3 below.

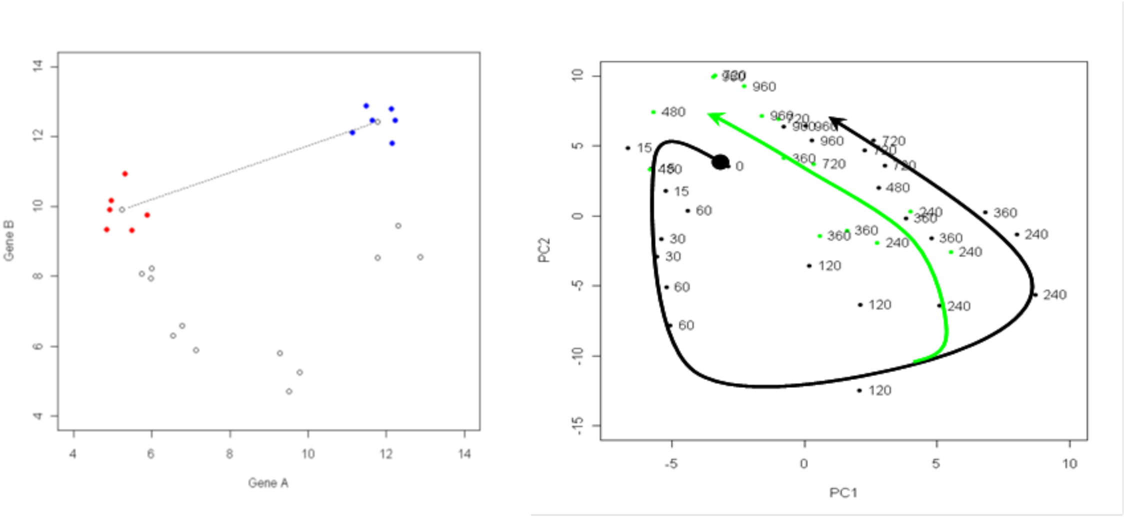

Additional Figure S3: Analysis of microarray time series by dimension reduction methods. Left panel shows synthetic time series with 10 time points showing the two first principal components. Red and blue dots indicate gene expression states at the start (e.g. adding of a stimulus) and at the end or steady state, respectively. A direct comparison of gene expression levels between the start and the end state (dotted line) will give a list of genes that have significantly changed their expression level between the two states. While following the change from start to end (grey arrows) the total number of comparisons increases with the square of number of time steps examined. Right panel shows a score plot indicating the time points from 15 min to 16 h. The straight lines show up- or down-regulation of the same genes (black: sustained gastrin stimulation; green transient gastrin stimulation) while corners indicate that new genes change their expression levels. This score plot shows that the gene transcript levels are down-regulated faster in cells subjected to transient gastrin treatment compared to cells with sustained gastrin treatment. See main text for additional information.

ADDITIONAL FILE 2: additional methods

cDNA Microarray procedure: cDNA Microarrays were manufactured by the NTNU microarray FUGE platform (http://www.microarray.no) using 15000 cDNA rat probes from Research Genetics (IMAGE collection) printed in duplicates on Corning CMT Gaps II slides (Corning Inc. NY). The probes were dissolved in 50% DMSO to ensure high printing quality. The microarray slides were UV cross linked at 300 mJ in order to fix the DNA to the glass slides.

Labeling was performed by reverse transcription of total RNA (3 µg) in the presence of primers containing the capture sequence for subsequent hybridization of Cy3- and Cy5-labeled dendrimers, using the Genisphere 3DNA Array 350 Expression Array Detection kit (Genisphere, Hatfield, PA) as described in the manufacturer's protocol. Blocking procedures were performed as previously described [1]. Briefly, Mouse COT-1 DNA (Life Technologies) (0.1 µg/µg RNA) and 250 ng LNA dT Blocker (Genisphere, Hatfield, PA) were added to all mixtures in order to block hybridization of repetitive elements and reduce poly (dA) signal, respectively. Hybridization was performed in a humidified hybridization chamber (Corning Inc., NY) at 60°C for 14-15 hours in a total hybridization volume of 60 µl. Post hybridization washes were done with 2 × SSC and 0.2% SDS (15 min at 55°C), with 2 × SSC (10 minutes at room temperature), and finally with 2 × SSC

(10 minutes at room temperature). After the washing, the Cy3- and Cy5-labeled dendrimers were hybridized to the capture sequence at the reverse transcribed sample RNA at 60°C for 3 hours as described in the manufacturer's protocol. After dendrimer hybridization, the slides were washed as described above and dried by centrifugation at 1500 rpm for 5 minutes.

Scanning and image analysis of cDNA microarrays: The slides were scanned at a resolution of 10 µm by use of Packard Bioscience Scanarray Express HT scanner (Packard BioScience, Billerica, MA). A laser power of 100% was used, and excitation of Cy3 and Cy5 was performed at a wavelength of 532 nm and 635 nm, respectively. Signals were detected by use of photomultiplier tubes (PMTs) with two channels. In order to maximize the certainty of the weakest spots, the PMT voltage was adjusted to keep the background intensity between 200-400 (mean spot intensity) in each channel as suggested by Lyng and co-authors [4]. The GenePix 5.0 image analysis software (Axon Instruments, Inc., Union City, CA) was used for spot segmentation and intensity calculations. Spots and regions with high unspecific binding of dye or dust particles were manually flagged and excluded from the analysis.

Data analysis of cDNA microarrays: After removal of manually flagged spots, arrays were normalized using *loess* to remove intensity dependent variations in the ratios [5]. No background subtraction was performed as this had not increased the likelihood of finding differentially expressed genes with these arrays in a previous optimization study [1]. MA plots and image plots of the data were inspected, and 6 arrays that were not found to be of acceptable quality were removed. Differentially expressed genes between T0 and all other experimental conditions as well as between the gastrin treated conditions and the controls at each time point were found using a linear model with a Bayesian adjustment of variances [6]. p-values were corrected for multiple testing using the method of Benjamini and Hochberg [7] which produces a false discovery rate estimate (FDR), and genes with a corrected p-value below 0.05 were taken as significant

Database submission of microarray data: The microarray data were prepared according to minimum information about a microarray experiment (MIAME) recommendations [8] and deposited in the Array Express (http://www.ebi.ac.uk/microarray-as/ae/) [9]. Detailed information about the microarray designs and raw data files are accessible in ArrayExpress by use of these accession numbers: **E-MTAB-123**.

References:

1. Bruland T, Anderssen E, Doseth B, Bergum H, Beisvag V, Laegreid A: **Optimization of cDNA microarrays procedures using criteria that do not rely on external standards**. *BMC Genomics* 2007, **8**:377.
2. Androulakis IP, Yang E, Almon RR: **Analysis of time-series gene expression data: methods, challenges, and opportunities**. *Annu Rev Biomed Eng* 2007, **9**:205-228.
3. Gidskehaug L, Anderssen E, Flatberg A, Alsberg BK: **A framework for significance analysis of gene expression data using dimension reduction methods**. *BMC Bioinformatics* 2007, **8**:346.
4. Lyng H, Badiee A, Svendsrud DH, Hovig E, Myklebost O, Stokke T: **Profound influence of microarray scanner characteristics on gene expression ratios: analysis and procedure for correction**. *BMC Genomics* 2004, **5**(1):10.
5. Yang YH, Dudoit S, Luu P, Lin DM, Peng V, Ngai J, Speed TP: **Normalization for cDNA microarray data: a robust composite method addressing single and multiple slide systematic variation**. *Nucl Acids Res* 2002, **30**(4):e15-.

6. Smyth GK: **Linear models and empirical bayes methods for assessing differential expression in microarray experiments**. *Stat Appl Genet Mol Biol* 2004, **3**:Article3.
7. Benjamini Y, Hochberg Y: **Controlling the False Discovery Rate - a Practical and Powerful Approach to Multiple Testing**. *Journal of the Royal Statistical Society Series B-Methodological* 1995, **57**(1):289-300.
8. Brazma A, Hingamp P, Quackenbush J, Sherlock G, Spellman P, Stoeckert C, Aach J, Ansorge W, Ball CA, Causton HC *et al*: **Minimum information about a microarray experiment (MIAME)-toward standards for microarray data**. *Nat Genet* 2001, **29**(4):365-371.
9. Parkinson H, Sarkans U, Shojatalab M, Abeygunawardena N, Contrino S, Coulson R, Farne A, Lara GG, Holloway E, Kapushesky M *et al*: **ArrayExpress--a public repository for microarray gene expression data at the EBI**. *Nucleic Acids Res* 2005, **33**(Database issue):D553-555.
