# Supplementary material for: The duration of gastrin treatment affects global gene expression and molecular responses involved in ER stress and anti-apoptosis
Source: BMC Genomics. 2013 Jun 28;14:429. doi: 10.1186/1471-2164-14-429 (PMC3698217; doi:10.1186/1471-2164-14-429)
Supplement: Additional file 3: Table S2 — Enrichment analysis of 259 genes significantly lower expressed in transiently versus sustained gastrin treated cells. Table S3: Enrichment analysis of 144 genes significantly higher expressed in transiently versus sustained gastrin treated cells. All 403 genes are shown in the heat map in Figure 1B and Additional file 1. [file 1471-2164-14-429-S3.pdf]

ADDITIONAL FILE 3

TABLE S2: Enrichment analysis of genes lower expressed in transiently *versus* sustained gastrin treated cells.

Enrichment by GO Processes	P-Values	Differentially expressed genes
Response to topologically incorrect protein	3,561E-15	ATF-4, ERp44, SERP1, FUS/DDIT3 fusion protein, DNAJB11, GRP78, C/EBP zeta, HSP70, Calreticulin, DnaJB9, ASNS, ATF-3, CHAC1, HERP, GFPT1, DNAJC3, ARMET, ERP5, SELS, Clusterin
Cellular amine metabolic process	4,056E-15	ATF-4, SERB, CDS1, GAD1, NARS, ARG2, SERA, SLC7A6, mini-TyrRS (IL8-like), AADAT, LARS1, TyrRS, GCH1, DNMT3A, CDP-diacylglycerol synthase, PDE, ASNS, SerRS, FARSB, AUMH, PDE1, rBAT, BCAT2, IleRS, ALT1, OAT, GSTM1_MOUSE, GFPT1, CBS, ASSY, SYTC, MAT1A, SLC7A5, Taspase1
Response to unfolded protein	2,288E-14	ATF-4, ERp44, SERP1, FUS/DDIT3 fusion protein, DNAJB11, GRP78, C/EBP zeta, HSP70, Calreticulin, DnaJB9, ASNS, ATF-3, CHAC1, HERP, GFPT1, DNAJC3, ARMET, ERP5, SELS
Activation of signaling protein activity involved in unfolded protein response	4,903E-14	ATF-4, SERP1, FUS/DDIT3 fusion protein, DNAJB11, GRP78, C/EBP zeta, HSP70, Calreticulin, DnaJB9, ASNS, ATF-3, HERP, GFPT1, DNAJC3, ERP5
Cellular amino acid metabolic process	5,931E-14	ATF-4, SERB, GAD1, NARS, ARG2, SERA, SLC7A6, mini-TyrRS (IL8-like), AADAT, LARS1, TyrRS, DNMT3A, ASNS, SerRS, FARSB, AUMH, rBAT, BCAT2, IleRS, ALT1, OAT, GSTM1_MOUSE, GFPT1, CBS, ASSY, SYTC, MAT1A, SLC7A5, Taspase1
Cellular response to unfolded protein/ Endoplasmic reticulum unfolded protein response	7,630E-14	ATF-4, SERP1, FUS/DDIT3 fusion protein, DNAJB11, GRP78, C/EBP zeta, HSP70, Calreticulin, DnaJB9, ASNS, ATF-3, HERP, GFPT1, DNAJC3, ERP5, SELS

Enrichment analysis of 259 genes significantly (Limma p<0.05) lower expressed in transiently *versus* sustained gastrin treated cells as shown in the heat map in Figure 1B and listed in Additional file 1. Enrichment analysis was performed using the MetaCore tool of GeneGo package http://www.genego.com, as described in Material and Methods.

ADDITIONAL FILE 3

TABLE S3: Enrichment analysis of genes higher expressed in transiently *versus* sustained gastrin treated cells.

Enrichment by GO Processes	P-Values	Differentially expressed genes
mitotic cell cycle	3,154E-08	Kid, PRC1, PCNA, VRK1, C15orf23, Wee1, NudE, NUSAP1, Cyclin B, GADD45 alpha, Cyclin B2, TTK, Stathmin, Cyclin A1, CDCA2, POLE2, MKLP1, Cyclin A, CDK1 (p34), 14-3-3, Dynamin
cell cycle process	1,475E-07	Kid, PRC1, PCNA, VRK1, C15orf23, Wee1, NudE, AMPK gamma subunit, NUSAP1, Cyclin B, GADD45 alpha, Cyclin B2, TTK, Stathmin, Cyclin A1, CDCA2, POLE2, Phosphatase regulator (inhibitor), MKLP1, Cyclin A, CDK1 (p34), 14-3-3, AMPK gamma2, Dynamin
G2/M transition of mitotic cell cycle	1,913E-07	Wee1, NudE, Cyclin B, GADD45 alpha, Cyclin B2, Cyclin A1, Cyclin A, CDK1 (p34), 14-3-3, Dynamin
cell cycle phase	5,358E-07	Kid, PRC1, PCNA, VRK1, C15orf23, Wee1, NudE, NUSAP1, Cyclin B, GADD45 alpha, Cyclin B2, TTK, Stathmin, Cyclin A1, CDCA2, POLE2, MKLP1, Cyclin A, CDK1 (p34), 14-3-3, Dynamin
Mitosis / Nuclear division	6,461E-07	Kid, VRK1, C15orf23, Wee1, NudE, NUSAP1, Cyclin B, Cyclin B2, Cyclin A1, CDCA2, MKLP1, Cyclin A, CDK1 (p34)

Enrichment analysis of 144 genes significantly (Limma p<0.05) higher expressed in transiently *versus* sustained gastrin treated cells as shown in the heat map in Figure 1B and listed in Additional file 1. Enrichment analysis was performed using the MetaCore tool of GeneGo package http://www.genego.com/ webcite, as described in Material and Methods.
