# Supplementary material for: The duration of gastrin treatment affects global gene expression and molecular responses involved in ER stress and anti-apoptosis
Source: BMC Genomics. 2013 Jun 28;14:429. doi: 10.1186/1471-2164-14-429 (PMC3698217; doi:10.1186/1471-2164-14-429)
Supplement: Additional file 5 — Temporal profiles of gastrin-induced genes with differing expression patterns in transient versus sustained mode described in Figure 5and Additional file4: Table S4. Time profiles as log2 expression data from three independent time series experiments are shown for each individual gene. Upper panels: transiently or sustained treated cells; Middle panels: sustained treated or untreated control cells. Lower panels: gene expression in the presence and absence of the protein synthesis inhibitor CHX. [file 1471-2164-14-429-S5.pdf]

ADDITIONAL FILE 5

Temporal profiles of selected genes from the 181 subset described in Figure 5 and Table S4. The genes are shown in the table below (subset of Table S4), and are sorted according to the groups defined in Figure 5 and Table S4 (a-f).

Probe_Id	EntrezID	Gene Symbol	Temporale profiles	Evaluation by CHX
ILMN_1350784	24517	Junb	**a**	primary
ILMN_1368356	314322	Fos	**a**	primary
ILMN_1357312	60430	Mcl1	**b**	primary
ILMN_1368718	362196	Chac1	**c**	secondary
ILMN_1370868	85430	Herpud1	**c**	uncertain
ILMN_1371686	83785	Vegfa	**c**	secondary
ILMN_1375100	79255	Atf4	**c**	uncertain
ILMN_1349910	29467	Ddit3	**d**	uncertain
ILMN_1363083	25389	Atf3	**d**	primary
ILMN_1372068	84580	Hdac5	**d**	primary
ILMN_1351240	25617	Hspa5	**e**	secondary
ILMN_1362286	25262	Itpr1	**e**	primary
ILMN_1355287	362653	Ctrc	**f**	secondary
ILMN_1355715	362347	Prss3 (LOC362347)	**f**	secondary
ILMN_1362767	24854	Clu	**f**	primary
ILMN_1369322	24691	Prss1	**f**	secondary
ILMN_1364198	266758	Sec11c	**f**	secondary
ILMN_1364459	298567	Cela3b	**f**	secondary
ILMN_1370696	113947	Maged2	**f**	primary
ILMN_1370842	498398	Selm	**f**	secondary

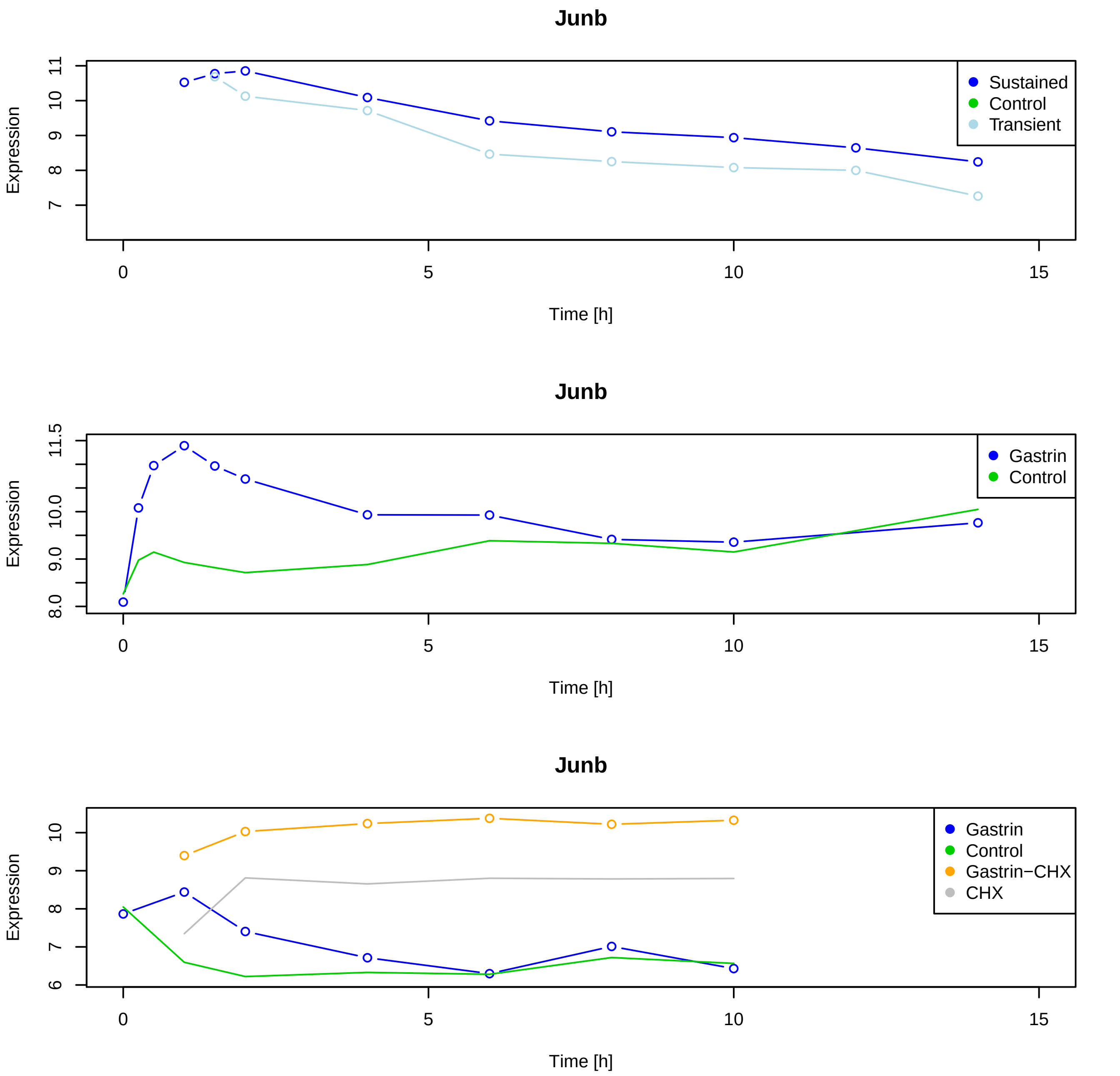
Junb
Expression
Time [h]
Sustained
Control
Transient
Junb
Expression
Time [h]
Gastrin
Control
Junb
Expression
Time [h]
Gastrin
Control
Gastrin−CHX
CHX

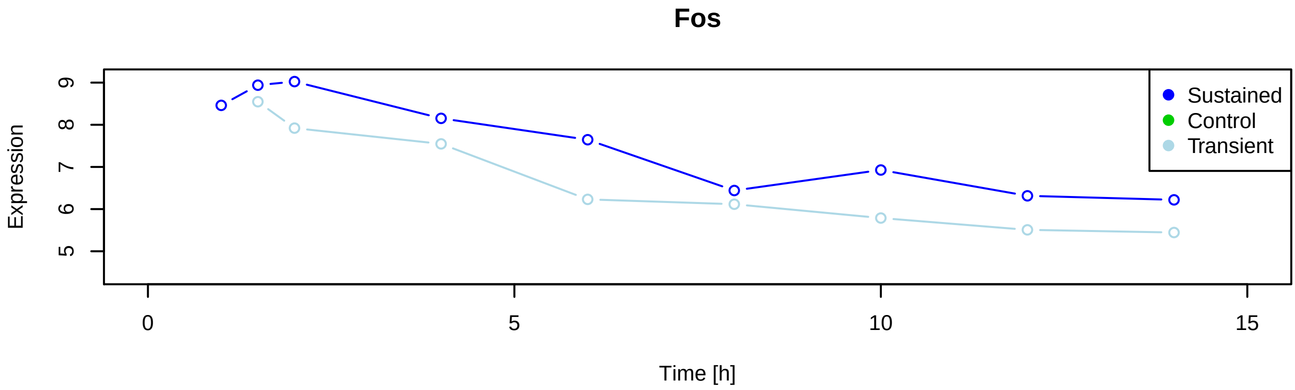
Fos
Sustained
Control
Transient
Expression
Time [h]

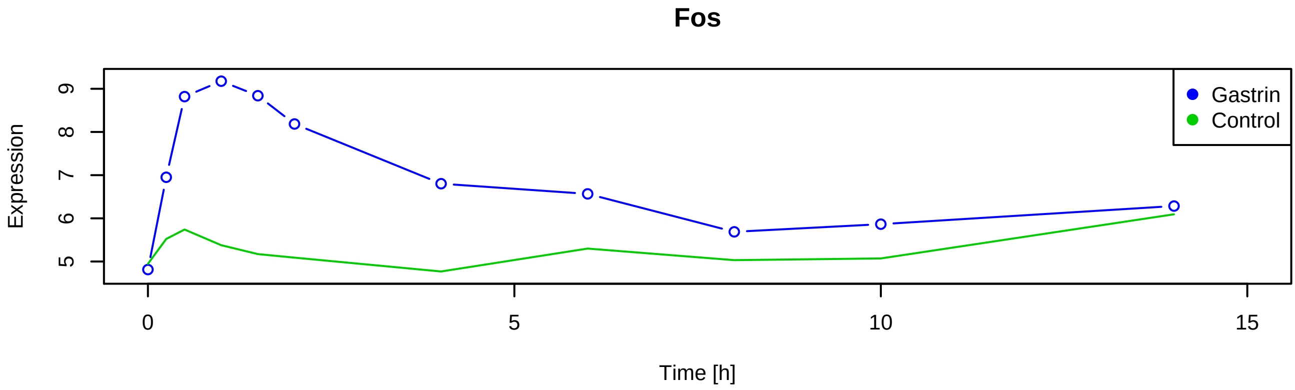
Fos
Gastrin
Control
Expression
Time [h]

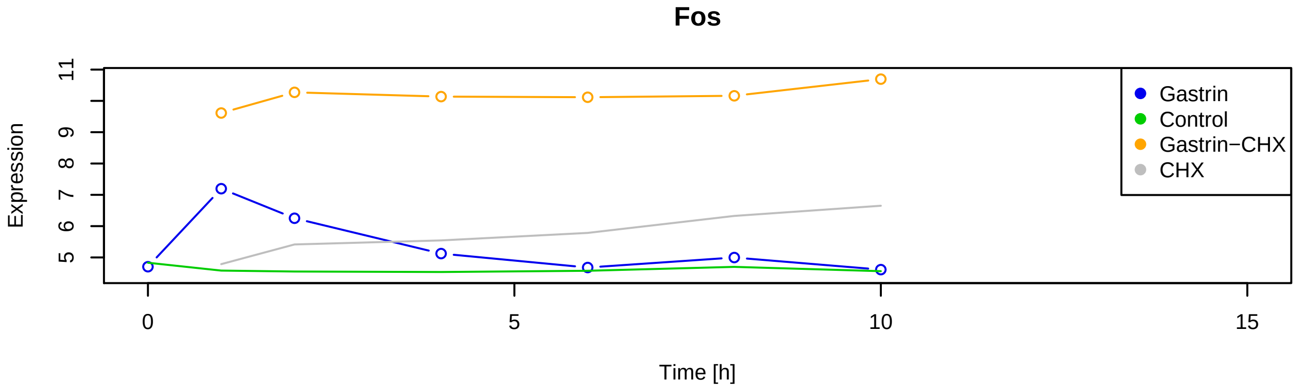
Fos
Gastrin
Control
Gastrin−CHX
CHX
Expression
Time [h]

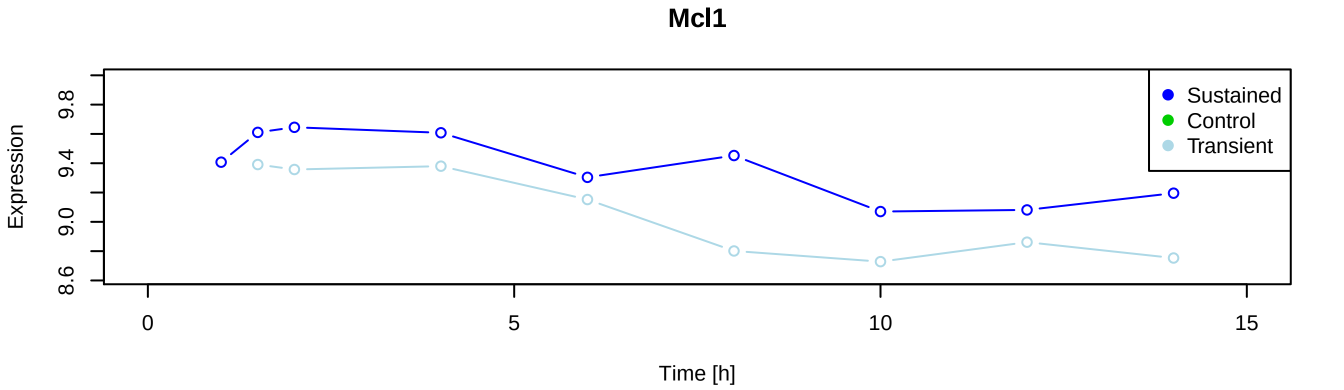
Mcl1
Expression
9.8
9.4
9.0
8.6
Sustained
Control
Transient
0
5
10
15
Time [h]

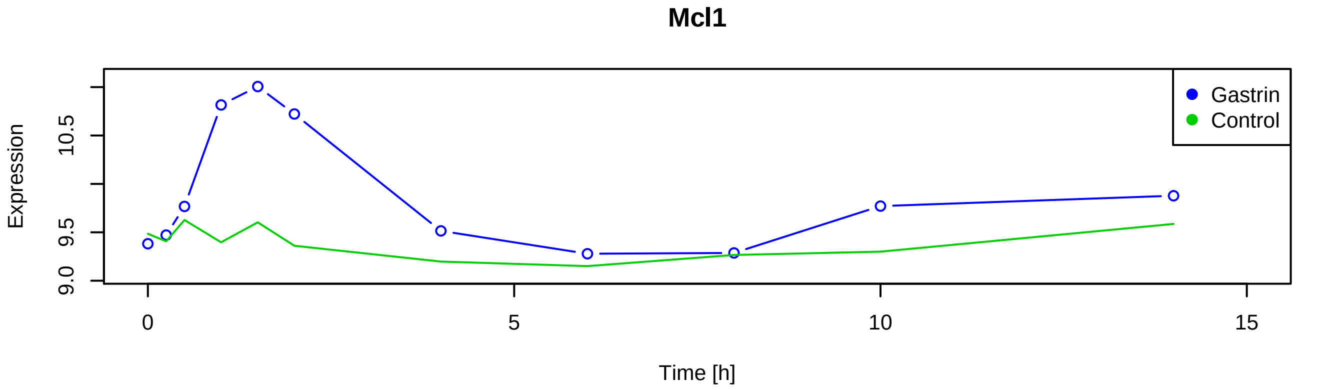
Mcl1
Expression
10.5
9.5
9.0
Gastrin
Control
0
5
10
15
Time [h]

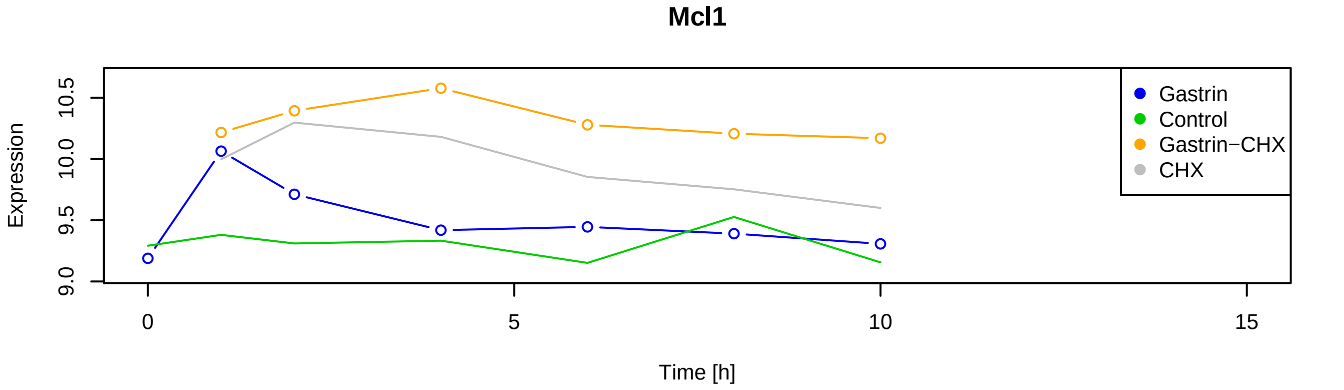
Mcl1
Expression
10.5
10.0
9.5
9.0
Gastrin
Control
Gastrin−CHX
CHX
0
5
10
15
Time [h]

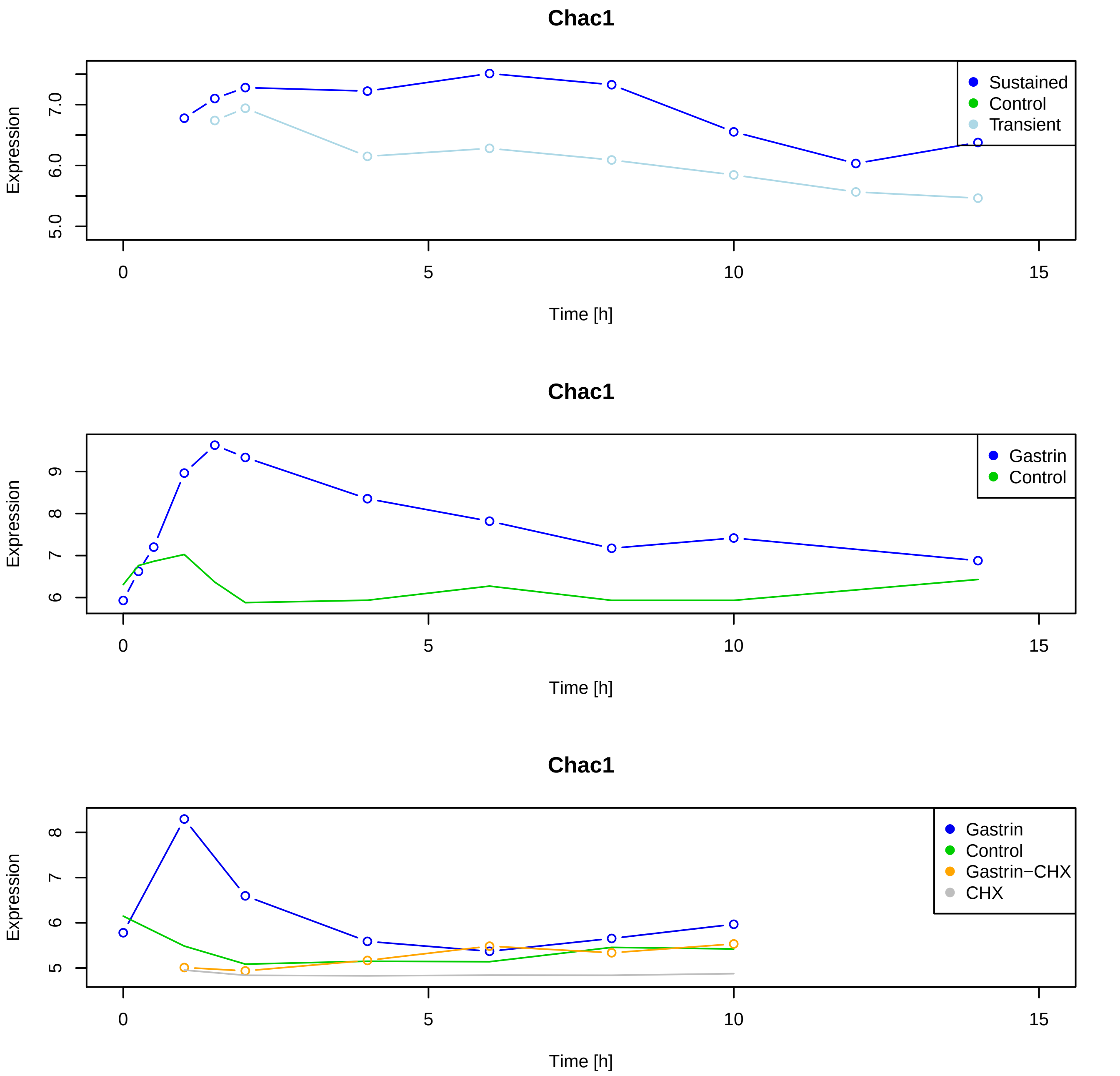
Chac1
Sustained
Control
Transient
Expression
5.0
6.0
7.0
0
5
10
15
Time [h]
Chac1
Gastrin
Control
Expression
6
7
8
9
0
5
10
15
Time [h]
Chac1
Gastrin
Control
Gastrin−CHX
CHX
Expression
5
6
7
8
0
5
10
15
Time [h]

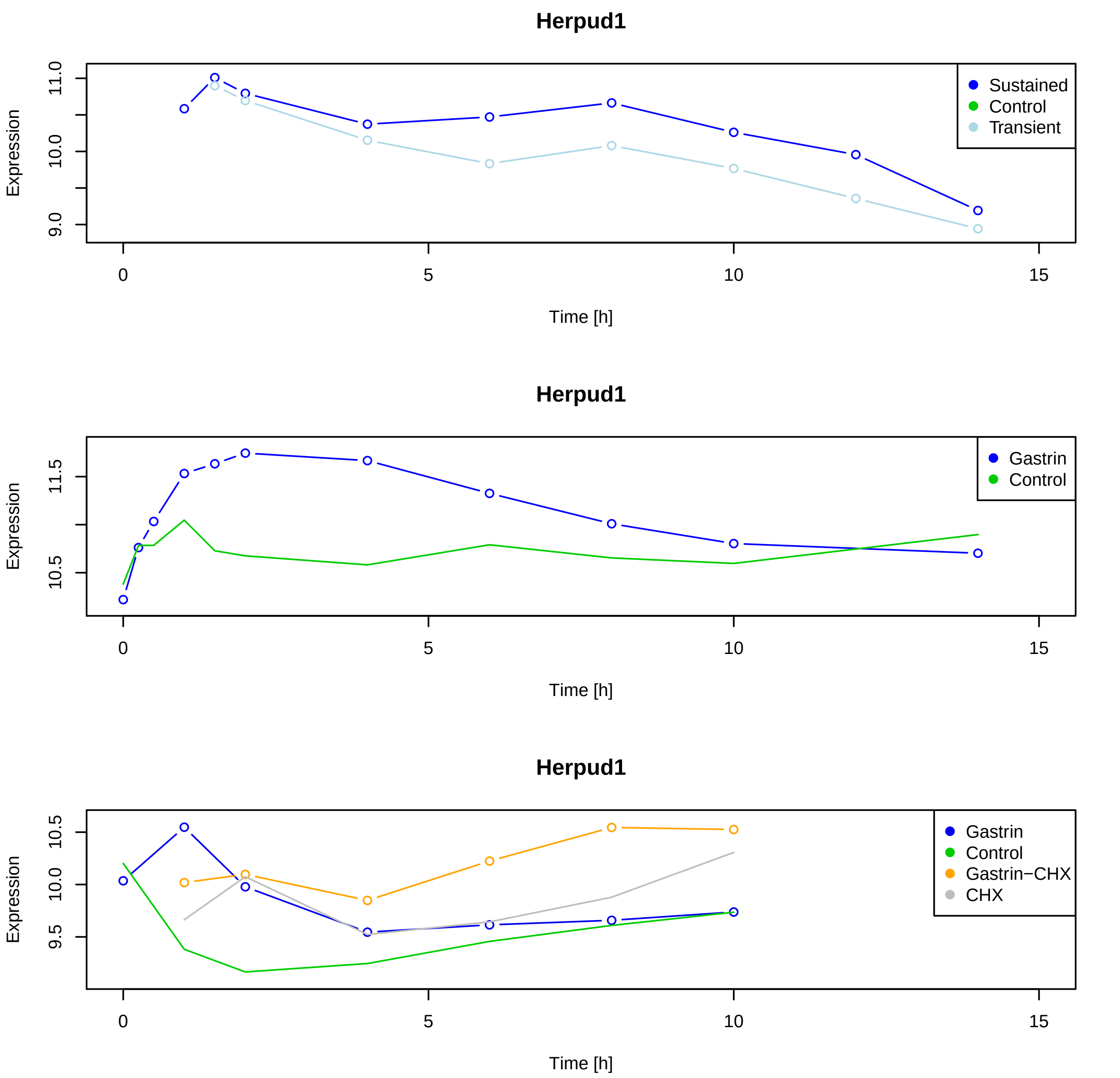
Herpud1
Expression
Time [h]
Sustained
Control
Transient
9.0
10.0
11.0
0
5
10
15
Herpud1
Expression
Time [h]
Gastrin
Control
10.5
11.5
0
5
10
15
Herpud1
Expression
Time [h]
Gastrin
Control
Gastrin−CHX
CHX
9.5
10.0
10.5
0
5
10
15

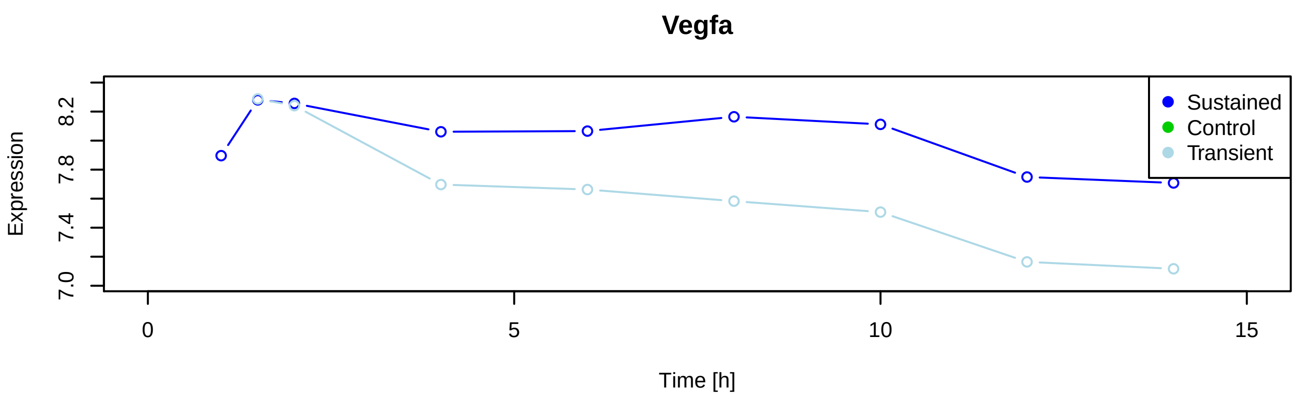
Vegfa
Expression
7.0
7.4
7.8
8.2
0
5
10
15
Time [h]
Sustained
Control
Transient

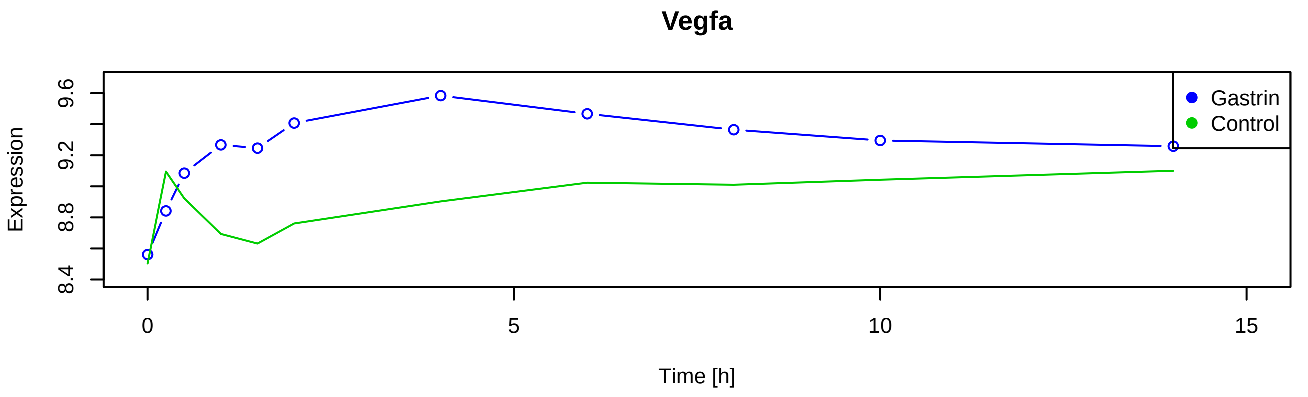
Vegfa
Expression
8.4
8.8
9.2
9.6
0
5
10
15
Time [h]
Gastrin
Control

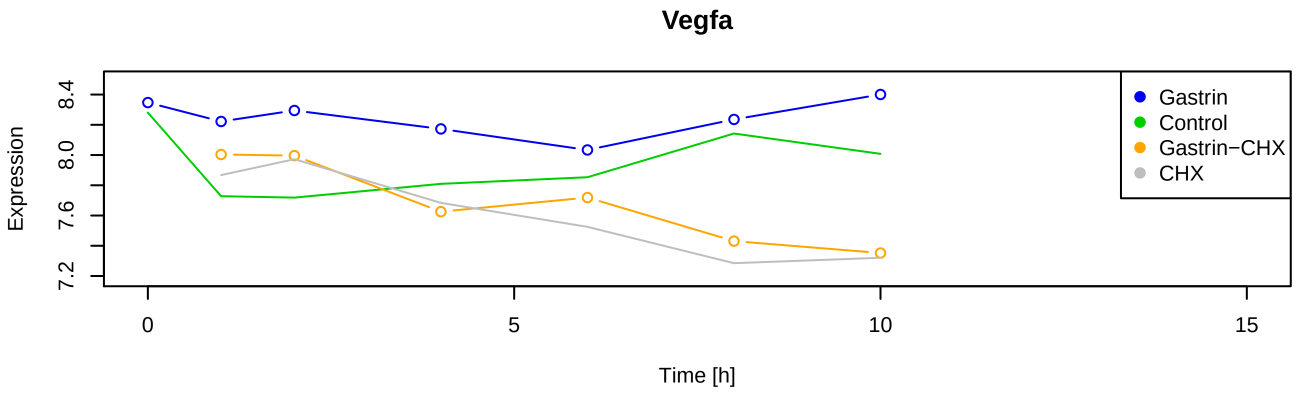
Vegfa
Expression
7.2
7.6
8.0
8.4
0
5
10
15
Time [h]
Gastrin
Control
Gastrin−CHX
CHX

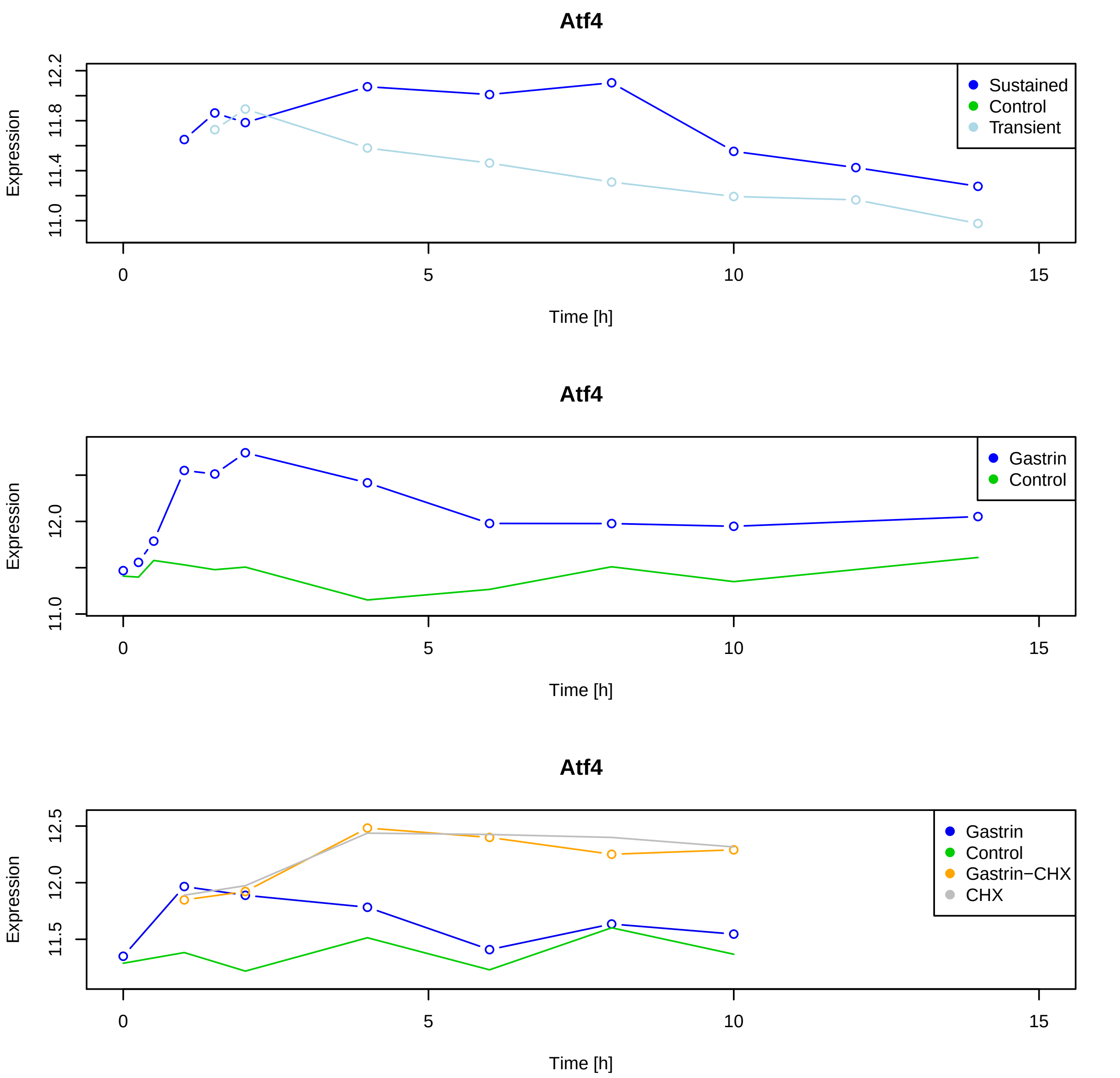
Atf4
Expression
Time [h]
Sustained
Control
Transient
Atf4
Expression
Time [h]
Gastrin
Control
Atf4
Expression
Time [h]
Gastrin
Control
Gastrin−CHX
CHX

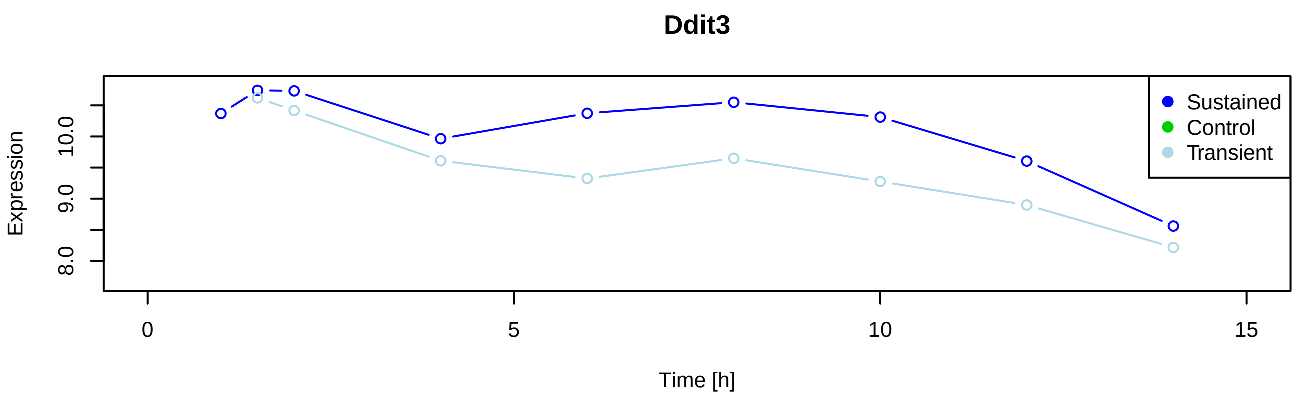
Ddit3
Expression
Time [h]
Sustained
Control
Transient
8.0
9.0
10.0
0
5
10
15

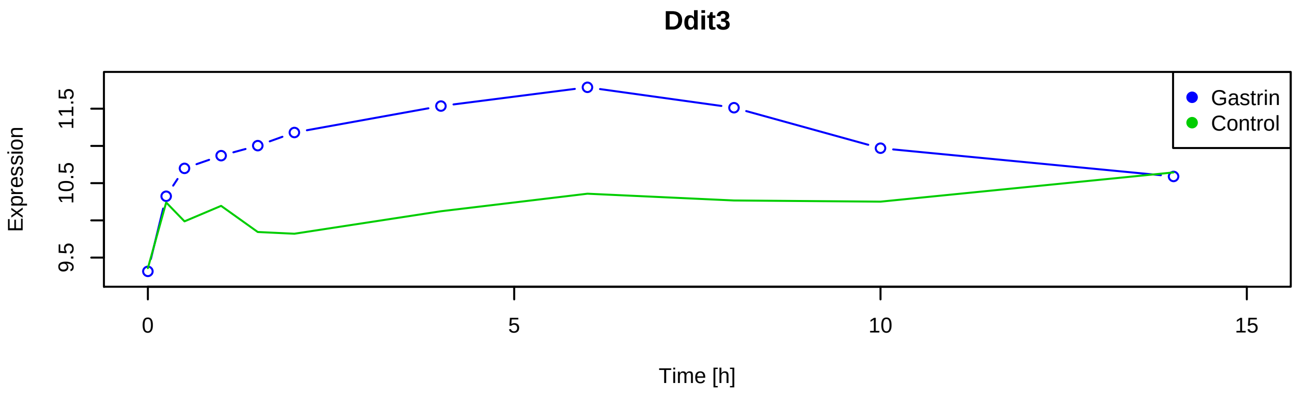
Ddit3
Expression
Time [h]
Gastrin
Control
9.5
10.5
11.5
0
5
10
15

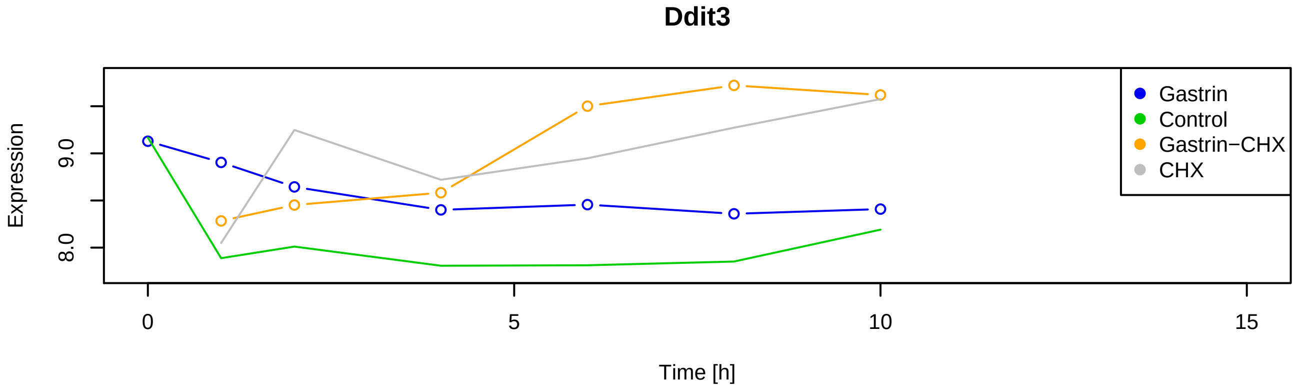
Ddit3
Expression
Time [h]
Gastrin
Control
Gastrin−CHX
CHX
8.0
9.0
0
5
10
15

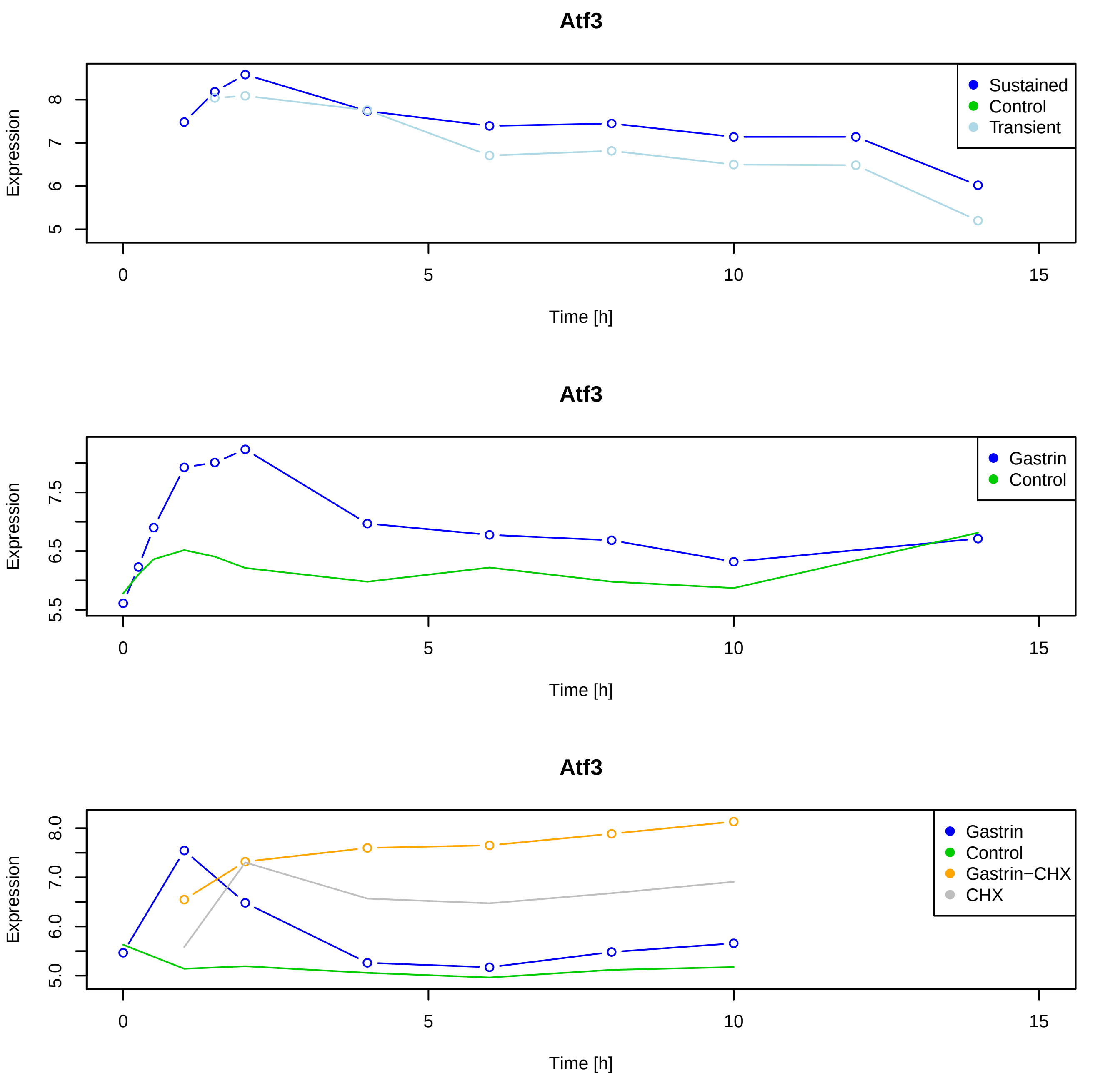

Atf3
Expression
8
7
6
5
0
5
10
15
Time [h]
Sustained
Control
Transient
Atf3
Expression
7.5
6.5
5.5
0
5
10
15
Time [h]
Gastrin
Control
Atf3
Expression
8.0
7.0
6.0
5.0
0
5
10
15
Time [h]
Gastrin
Control
Gastrin−CHX
CHX

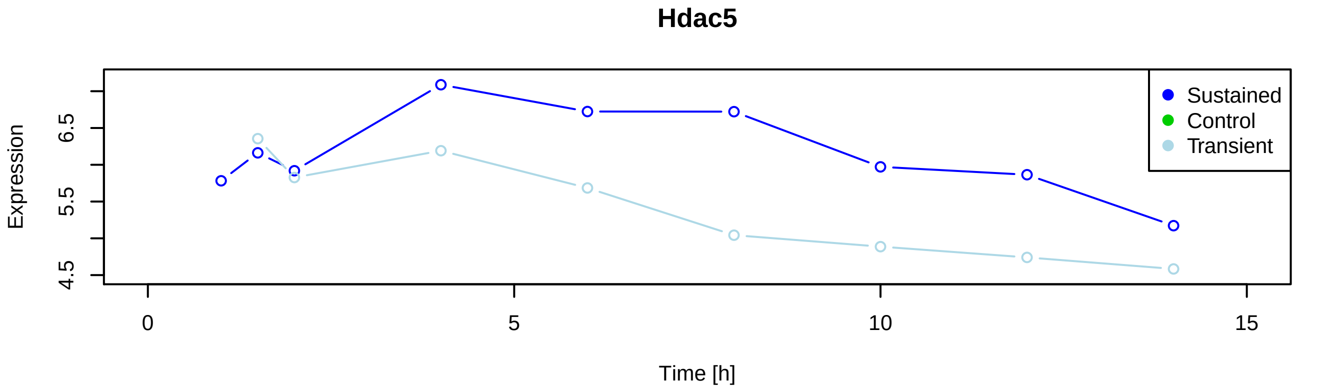
Hdac5
Expression
Time [h]
Sustained
Control
Transient

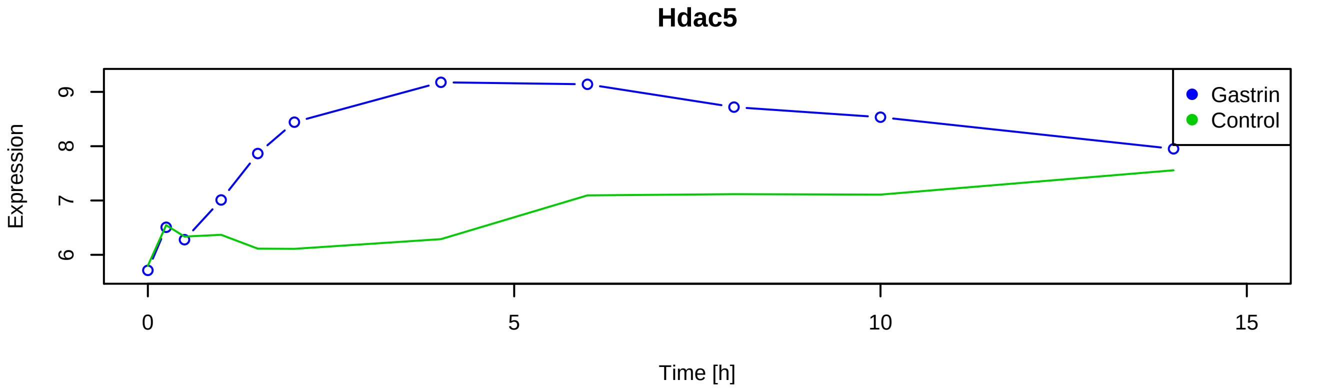
Hdac5
Expression
Time [h]
Gastrin
Control

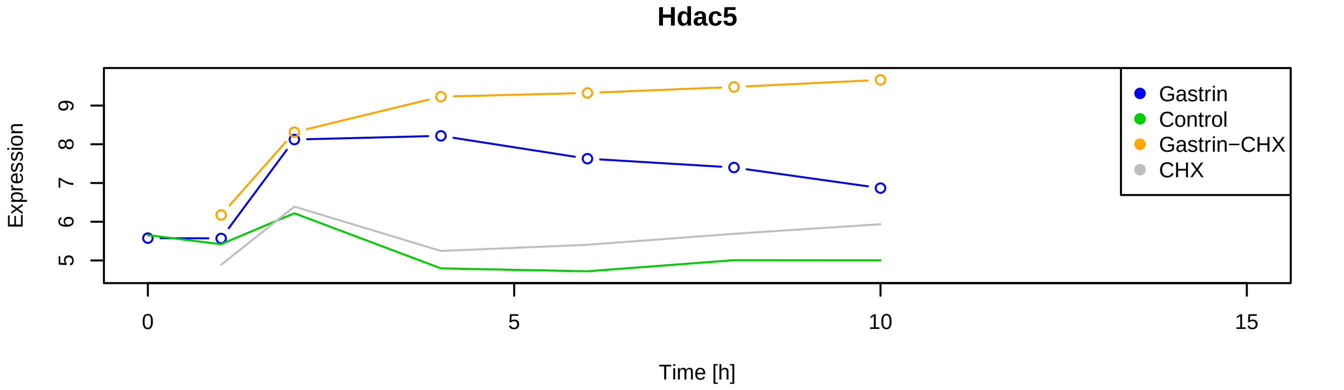
Hdac5
Expression
Time [h]
Gastrin
Control
Gastrin−CHX
CHX

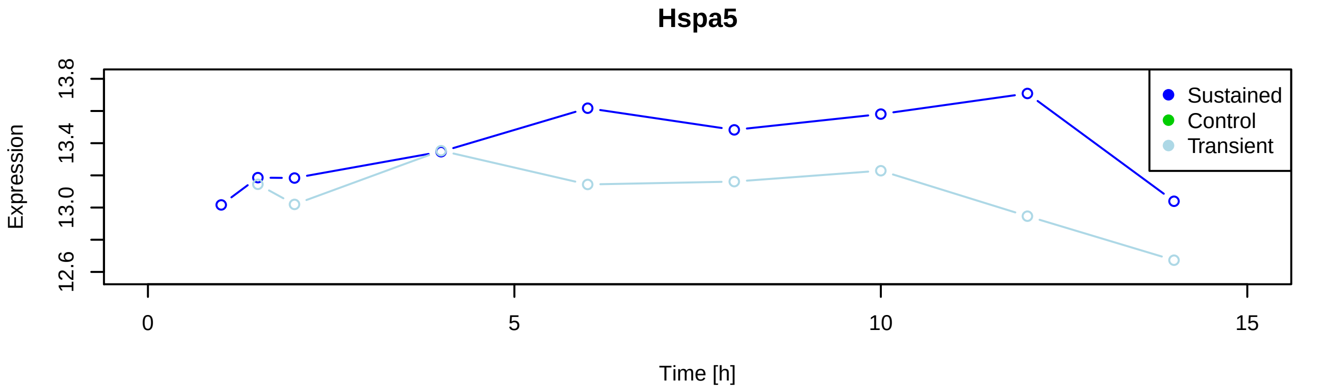
Hspa5
Expression
Time [h]
Sustained
Control
Transient
12.6
13.0
13.4
13.8
0
5
10
15

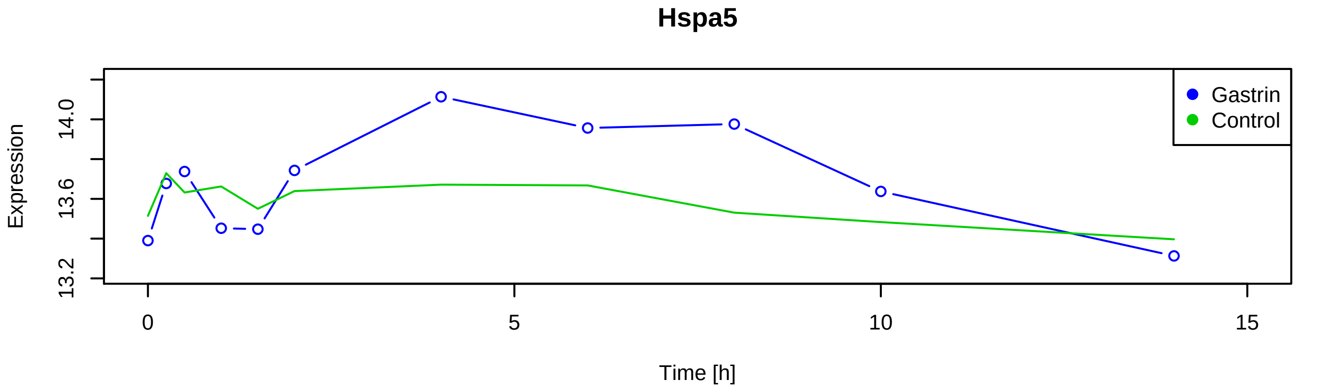
Hspa5
Expression
Time [h]
Gastrin
Control
13.2
13.6
14.0
0
5
10
15

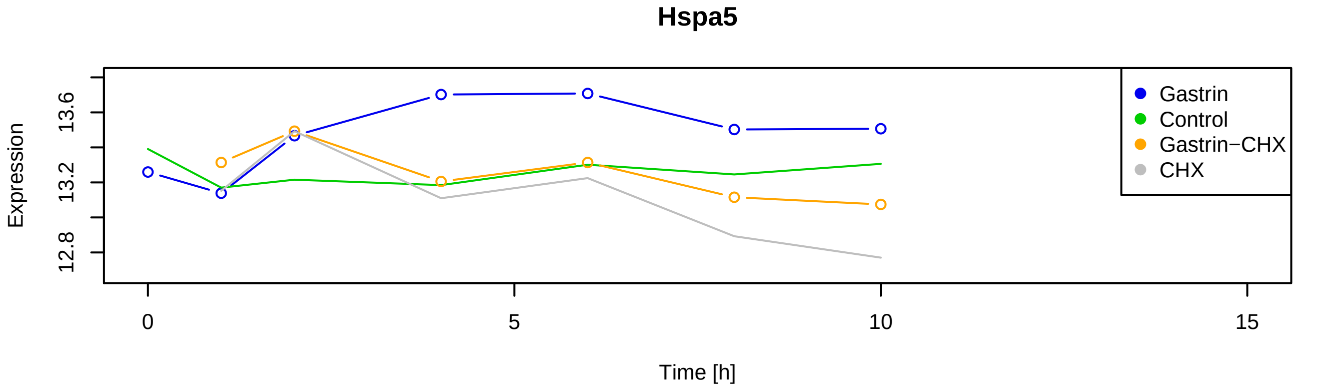
Hspa5
Expression
Time [h]
Gastrin
Control
Gastrin−CHX
CHX
12.8
13.2
13.6
0
5
10
15

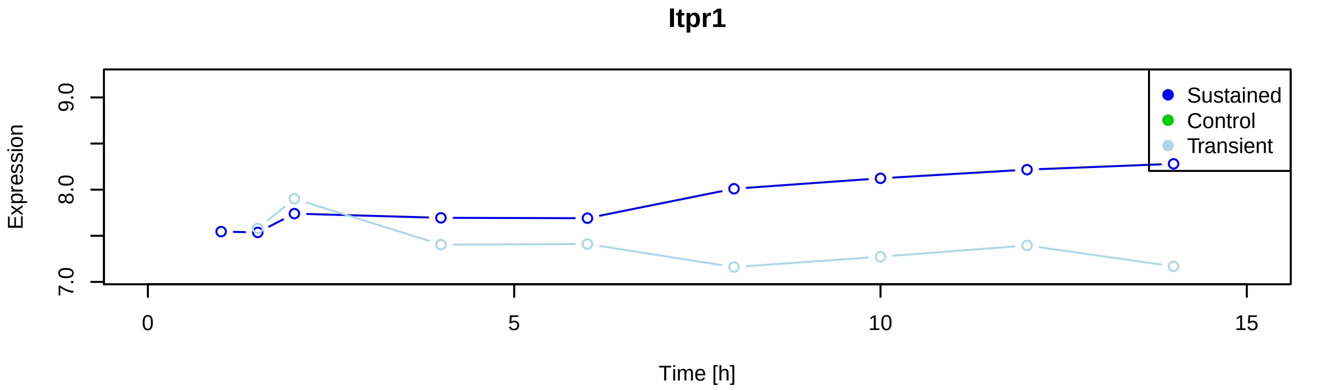
Itpr1
Expression
Time [h]
Sustained
Control
Transient
7.0
8.0
9.0
0
5
10
15

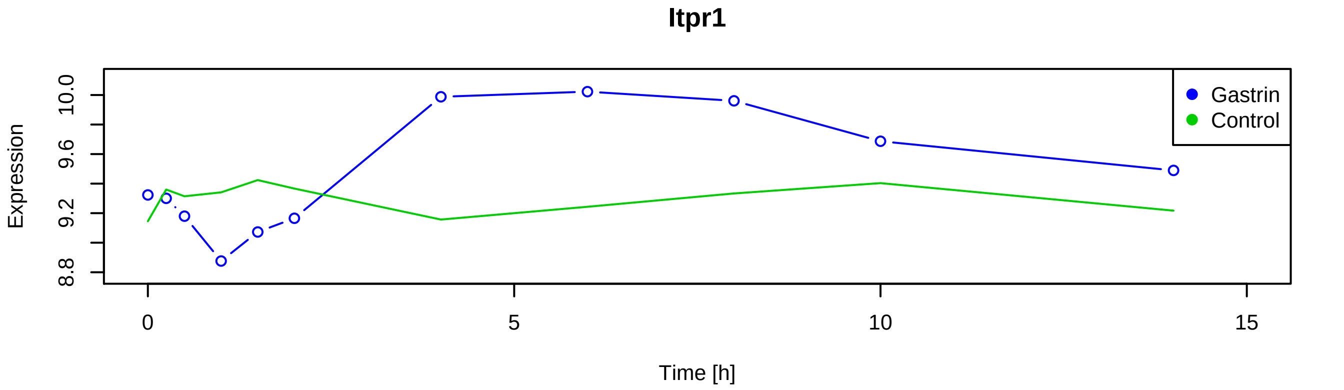
Itpr1
Expression
Time [h]
Gastrin
Control
8.8
9.2
9.6
10.0
0
5
10
15

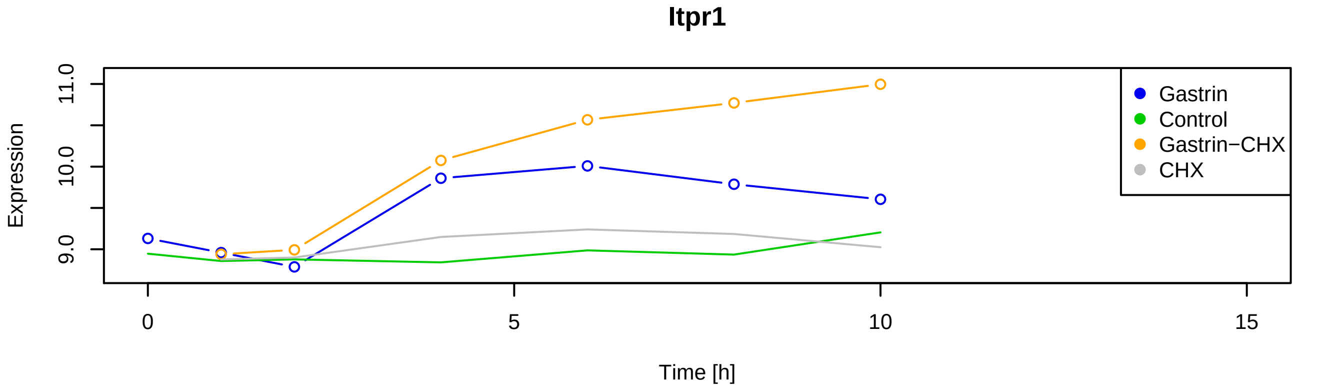
Itpr1
Expression
Time [h]
Gastrin
Control
Gastrin−CHX
CHX
9.0
10.0
11.0
0
5
10
15

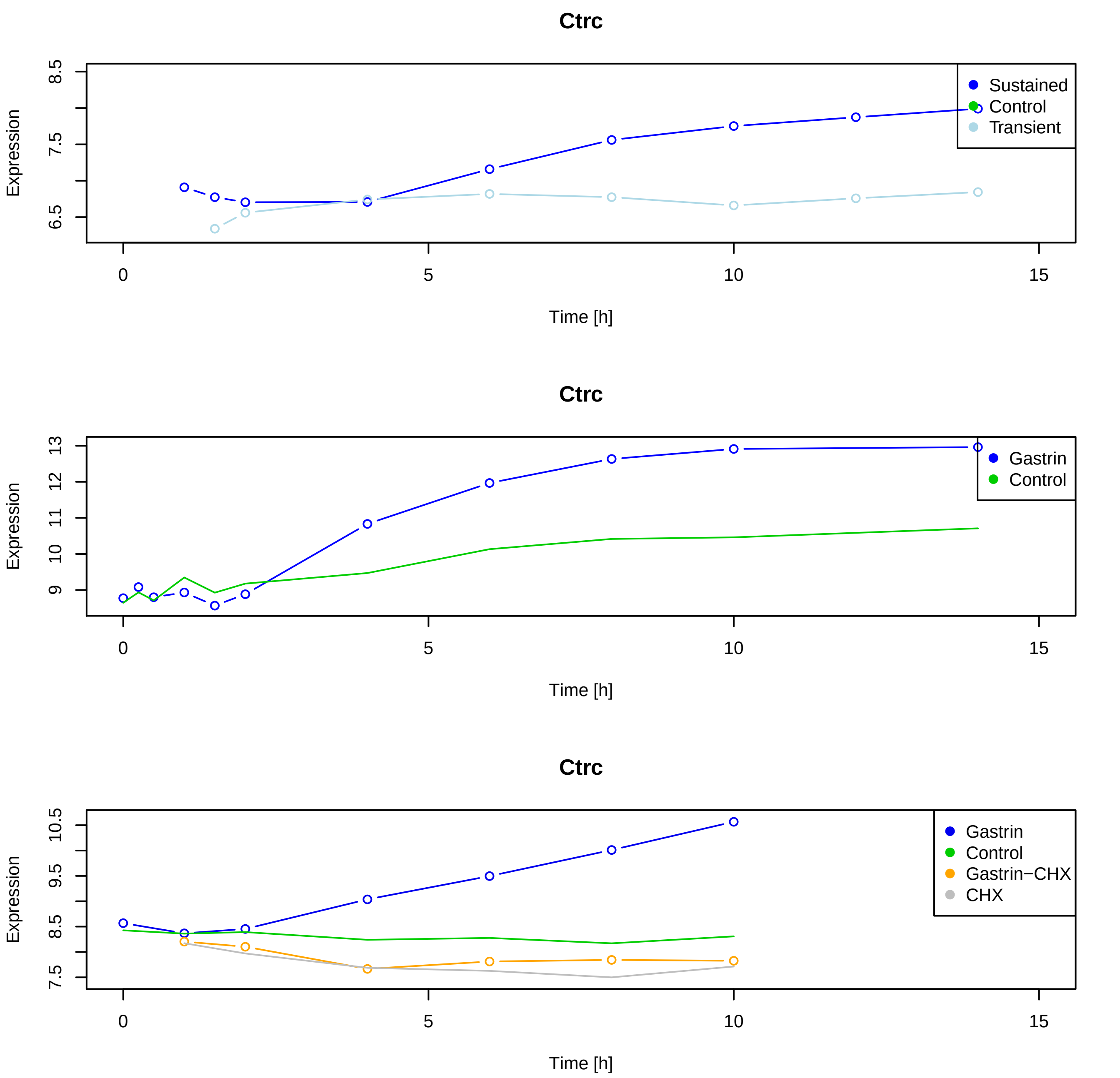

Ctrc
Expression
Time [h]
Sustained
Control
Transient
Ctrc
Expression
Time [h]
Gastrin
Control
Ctrc
Expression
Time [h]
Gastrin
Control
Gastrin−CHX
CHX

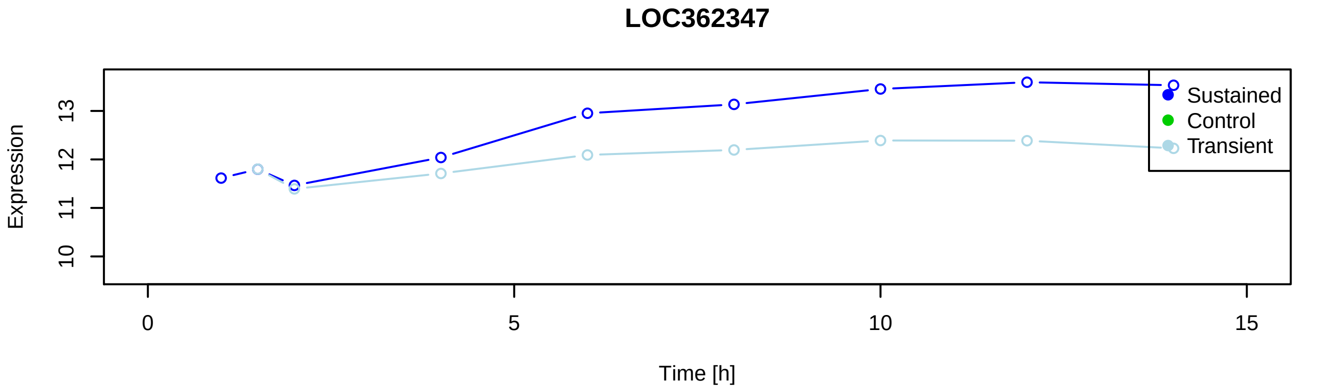
LOC362347
Expression
10
11
12
13
0
5
10
15
Time [h]
Sustained
Control
Transient

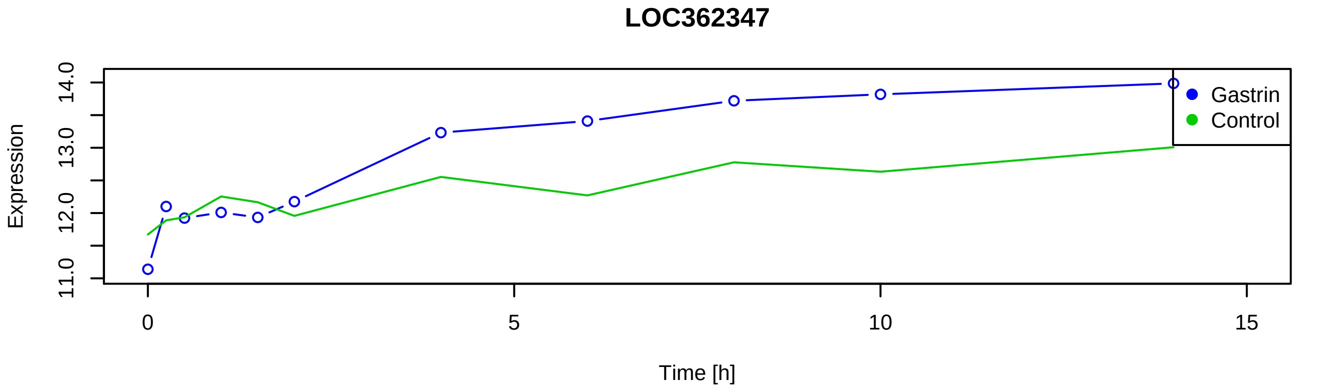
LOC362347
Expression
11.0
12.0
13.0
14.0
0
5
10
15
Time [h]
Gastrin
Control

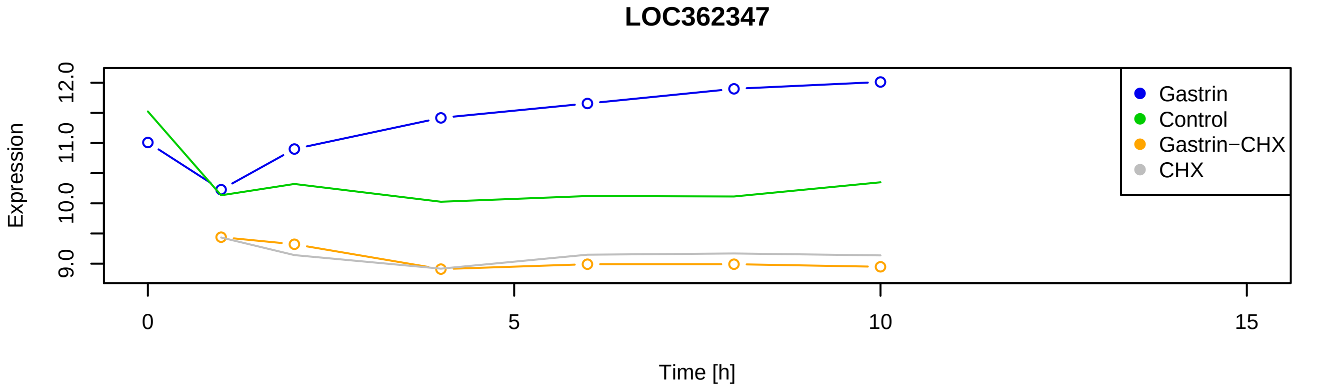
LOC362347
Expression
9.0
10.0
11.0
12.0
0
5
10
15
Time [h]
Gastrin
Control
Gastrin−CHX
CHX

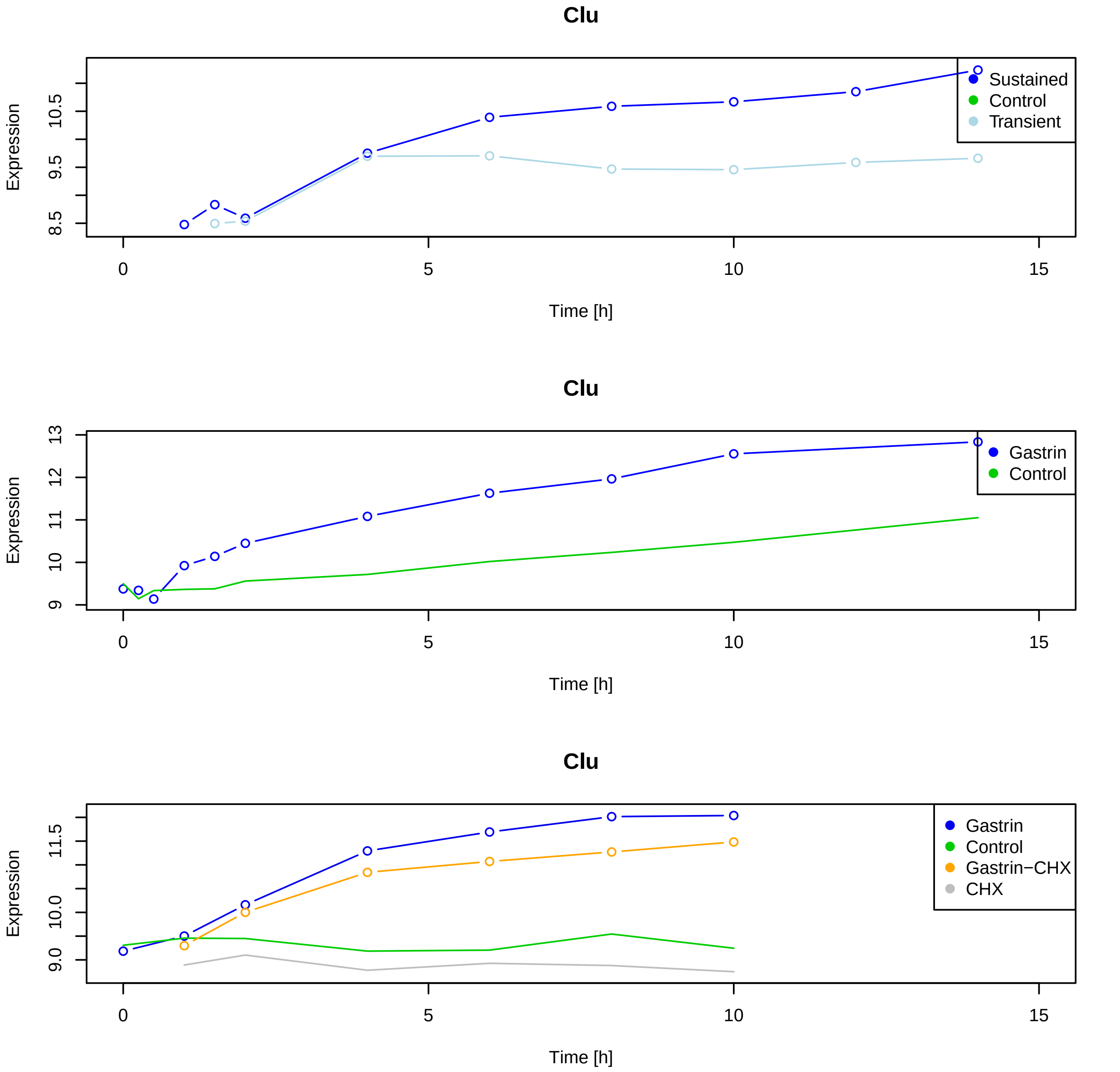
Clu
Expression
8.5
9.5
10.5
Sustained
Control
Transient
0
5
10
15
Time [h]
Clu
Expression
9
10
11
12
13
Gastrin
Control
0
5
10
15
Time [h]
Clu
Expression
9.0
10.0
11.5
Gastrin
Control
Gastrin−CHX
CHX
0
5
10
15
Time [h]

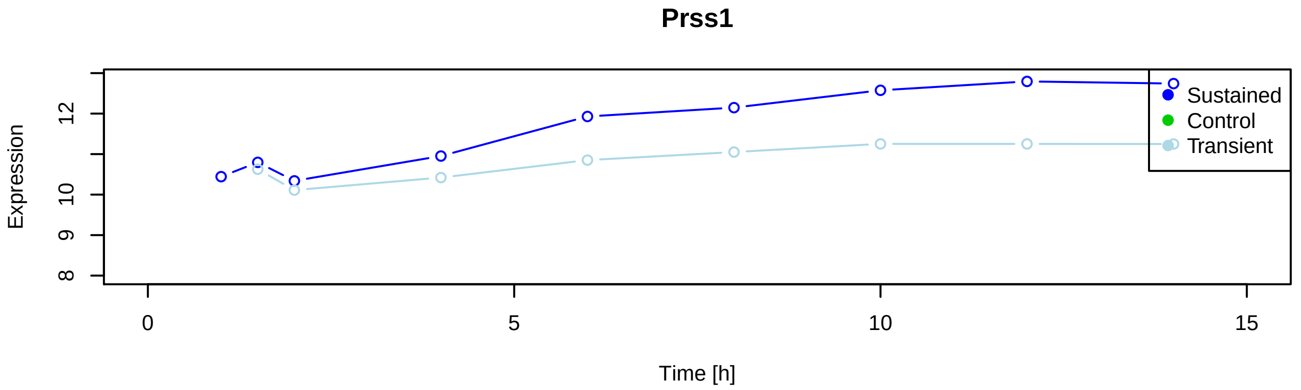
Prrs1
Expression
Time [h]
Sustained
Control
Transient
0
5
10
15
8
9
10
12

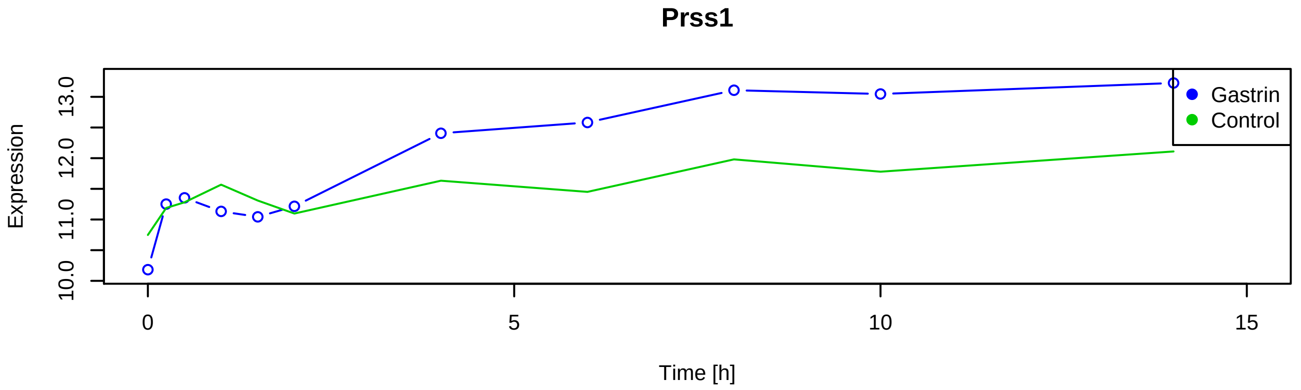
Prss1
Expression
Time [h]
Gastrin
Control
10.0
11.0
12.0
13.0
0
5
10
15

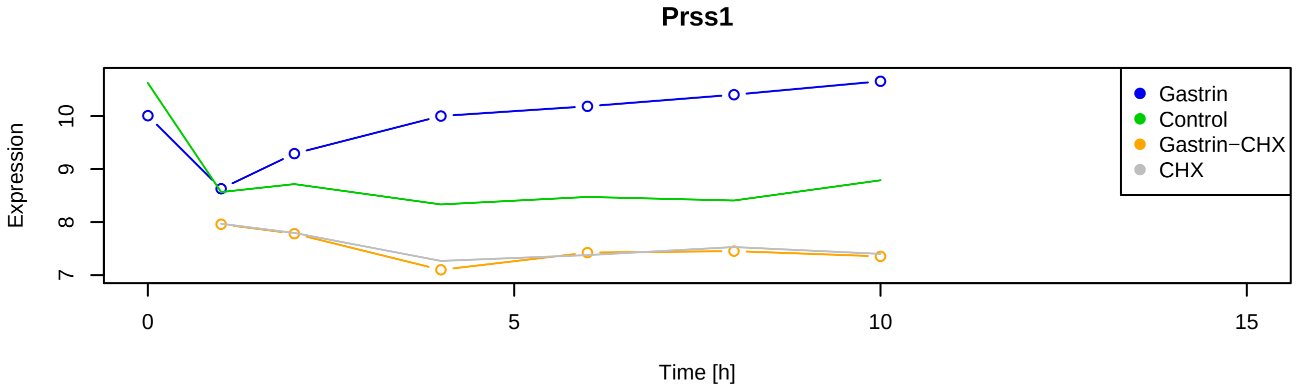
Prss1
Expression
Time [h]
Gastrin
Control
Gastrin−CHX
CHX
7
8
9
10
0
5
10
15

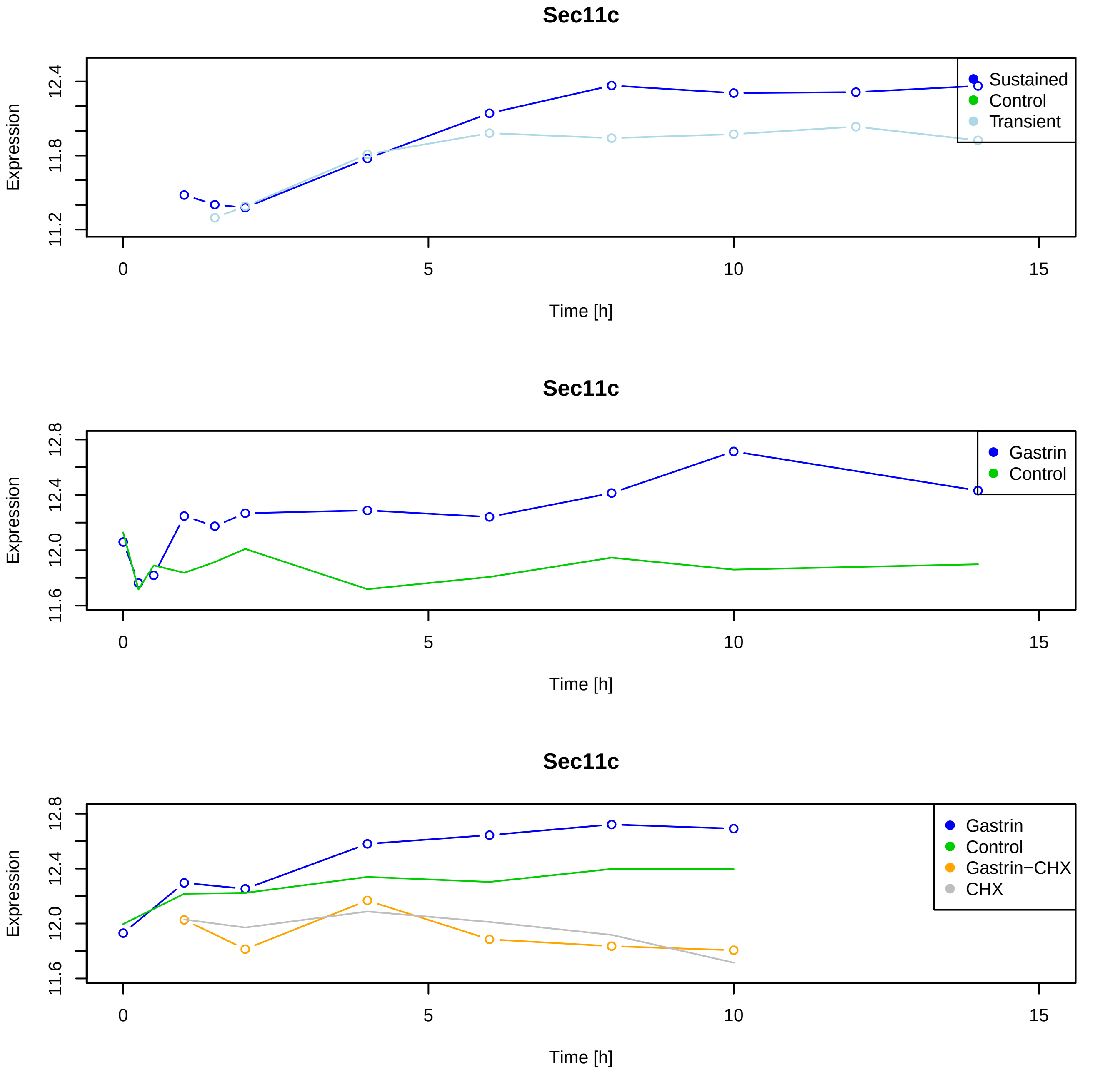
Sec11c
Expression
Time [h]
Sustained
Control
Transient
Sec11c
Expression
Time [h]
Gastrin
Control
Sec11c
Expression
Time [h]
Gastrin
Control
Gastrin−CHX
CHX

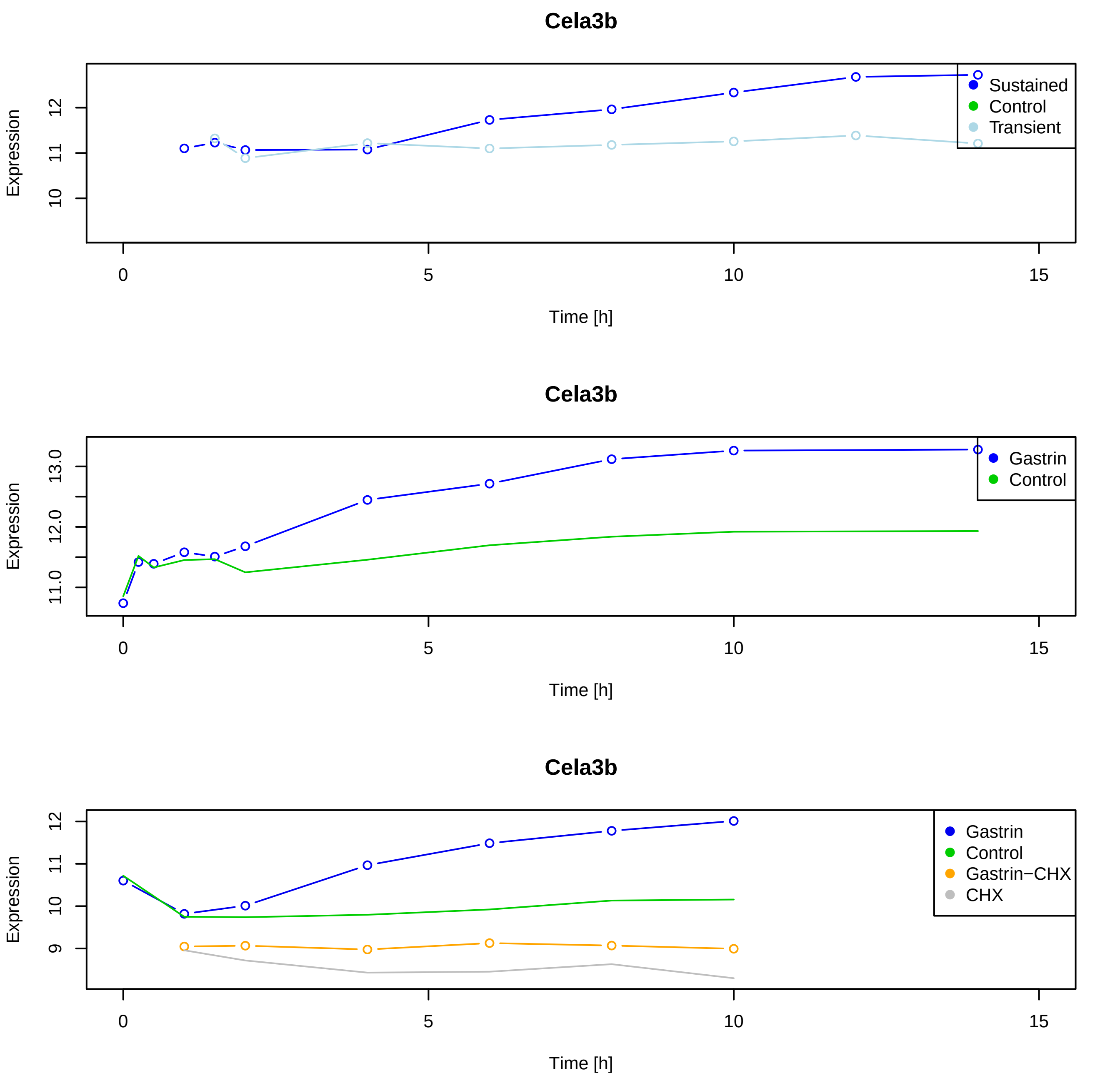
Cela3b
Sustained
Control
Transient
Expression
Time [h]
Cela3b
Gastrin
Control
Expression
Time [h]
Cela3b
Gastrin
Control
Gastrin−CHX
CHX
Expression
Time [h]

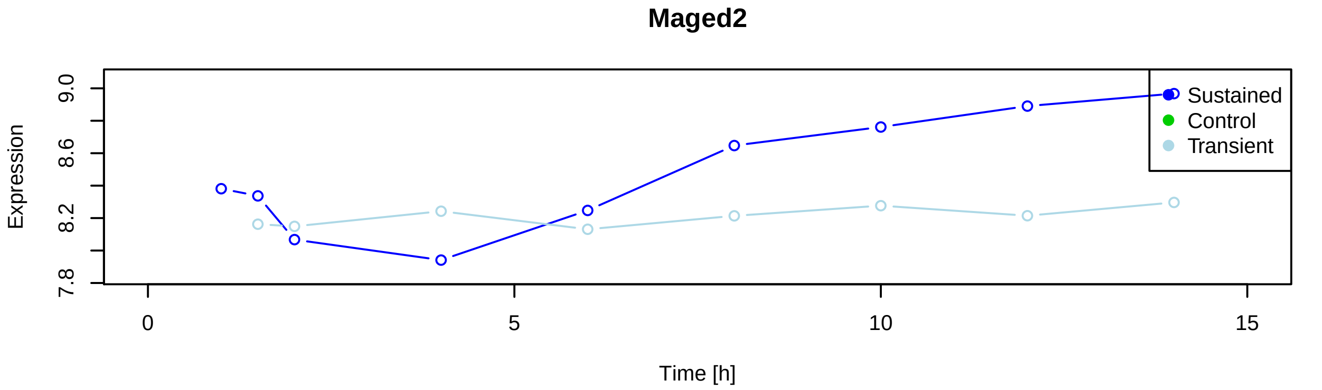

Maged2
Sustained
Control
Transient
Expression
7.8
8.2
8.6
9.0
0
5
10
15
Time [h]

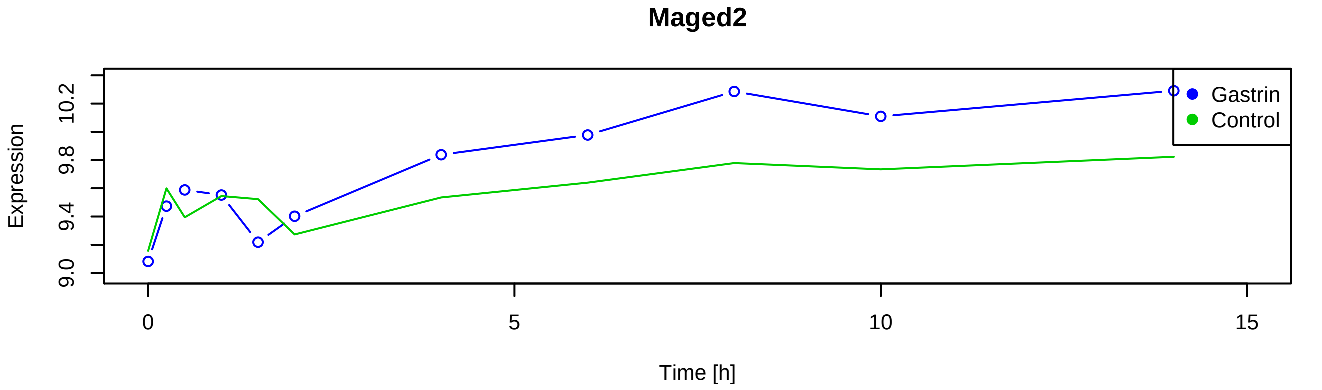

Maged2
Gastrin
Control
Expression
9.0
9.4
9.8
10.2
0
5
10
15
Time [h]

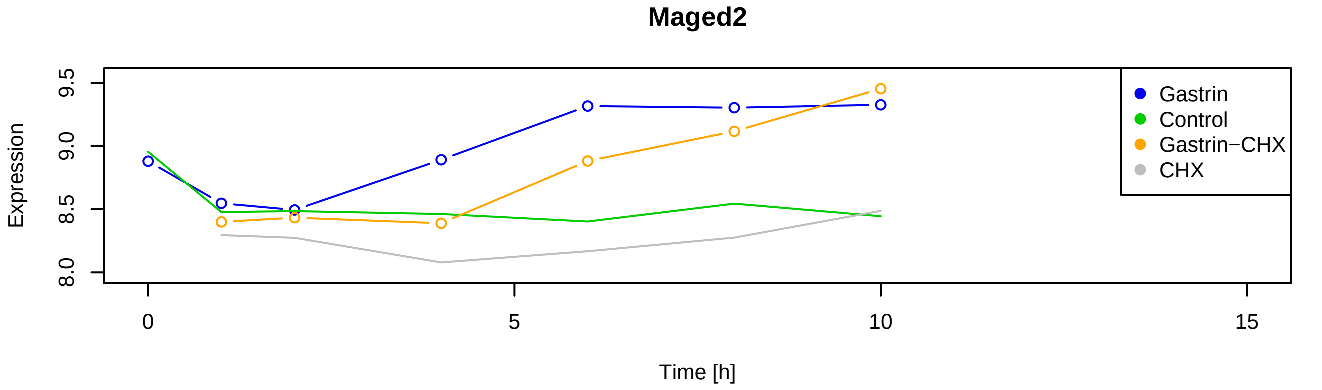

Maged2
Gastrin
Control
Gastrin−CHX
CHX
Expression
8.0
8.5
9.0
9.5
0
5
10
15
Time [h]

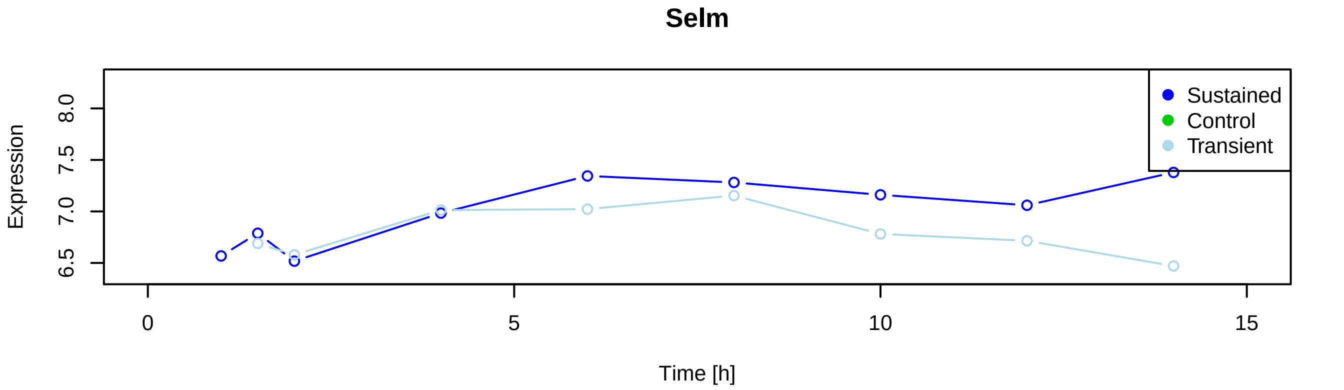
Selm
Sustained
Control
Transient
Expression
6.5
7.0
7.5
8.0
0
5
10
15
Time [h]

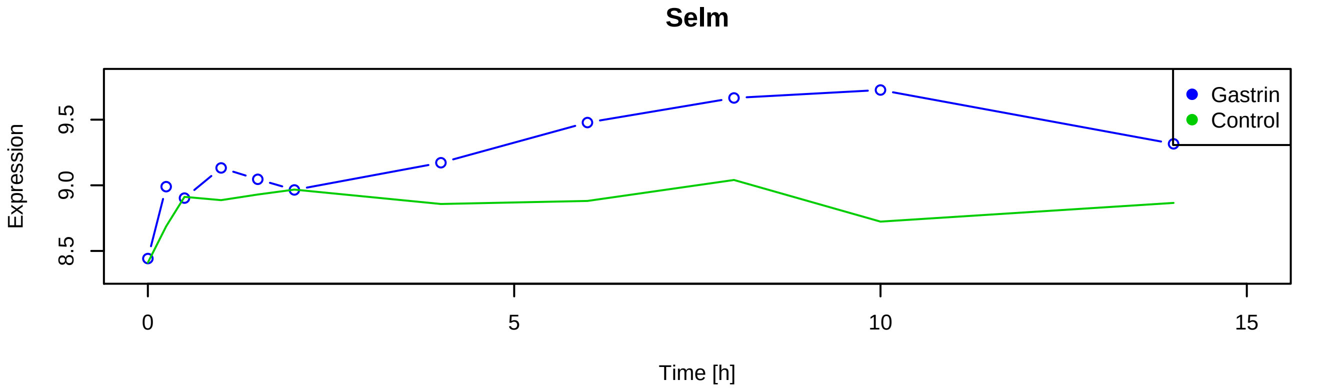
Selm
Gastrin
Control
Expression
8.5
9.0
9.5
0
5
10
15
Time [h]

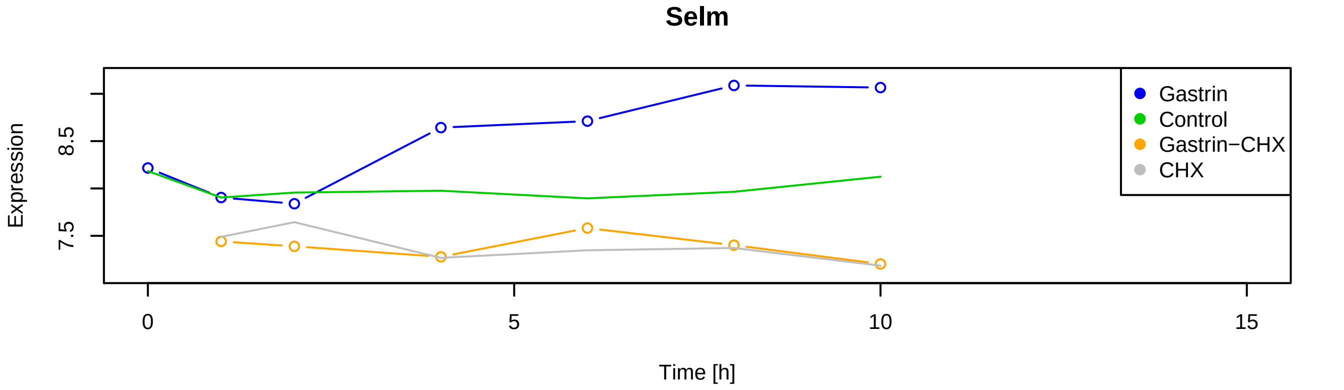
Selm
Gastrin
Control
Gastrin−CHX
CHX
Expression
7.5
8.5
0
5
10
15
Time [h]
